# A Numerically Subdominant CD8 T Cell Response to Matrix Protein of Respiratory Syncytial Virus Controls Infection with Limited Immunopathology

**DOI:** 10.1371/journal.ppat.1005486

**Published:** 2016-03-04

**Authors:** Jie Liu, Elias K. Haddad, Joshua Marceau, Kaitlyn M. Morabito, Srinivas S. Rao, Ali Filali-Mouhim, Rafick-Pierre Sekaly, Barney S. Graham

**Affiliations:** 1 Vaccine Research Center, National Institute of Allergy and Infectious Disease, National Institutes of Health, Bethesda, Maryland, United States of America; 2 Drexel University, Division of Infectious Diseases and HIV Medicine, Philadelphia, Pennsylvania, United States of America; 3 Department of Pathology, Case Western Reserve University, Cleveland, Ohio, United States of America; 4 Center for AIDS Research, Case Western Reserve University, Cleveland, Ohio, United States of America; St. Jude Children's Research Hospital, UNITED STATES

## Abstract

CD8 T cells are involved in pathogen clearance and infection-induced pathology in respiratory syncytial virus (RSV) infection. Studying bulk responses masks the contribution of individual CD8 T cell subsets to protective immunity and immunopathology. In particular, the roles of subdominant responses that are potentially beneficial to the host are rarely appreciated when the focus is on magnitude instead of quality of response. Here, by evaluating CD8 T cell responses in CB6F1 hybrid mice, in which multiple epitopes are recognized, we found that a numerically subdominant CD8 T cell response against D^b^M_187_ epitope of the virus matrix protein expressed high avidity TCR and enhanced signaling pathways associated with CD8 T cell effector functions. Each D^b^M_187_ T effector cell lysed more infected targets on a per cell basis than the numerically dominant K^d^M2_82_ T cells, and controlled virus replication more efficiently with less pulmonary inflammation and illness than the previously well-characterized K^d^M2_82_ T cell response. Our data suggest that the clinical outcome of viral infections is determined by the integrated functional properties of a variety of responding CD8 T cells, and that the highest magnitude response may not necessarily be the best in terms of benefit to the host. Understanding how to induce highly efficient and functional T cells would inform strategies for designing vaccines intended to provide T cell-mediated immunity.

## Author Summary

CD8 T cells play a key role in RSV clearance, immunopathology and disease. Therefore, CD8 T cells can help or harm the host depending on their timing, magnitude, and function. The CD8 T cell response represents a heterogeneous population of cells with phenotypically and functionally diverse subsets, and needs to at least be studied at the level of epitope specificity to understand how to diminish the risk of immunopathology. Studying the bulk response masks distinct contributions of individual CD8 T subsets to immunity and immunopathology. Focusing on CD8 T cell response with the highest magnitude overlooks role of subdominant responses. Here, we studied response to different epitopes and revealed that a numerically subdominant CD8 T cell response against D^b^M_187_ epitope of the virus matrix protein controlled virus replication efficiently with limited pulmonary inflammation and illness compared to the previously well-characterized and numerically dominant K^d^M2_82_ T cell response. Our data show that selectively boosting of epitope-specific CD8 T cell responses may be more beneficial than indiscriminant boosting of all available epitopes to achieve rapid viral clearance while limiting immunopathology. This work has implications for antigen design of vaccines intended to induce T-cell-mediated immunity.

## Introduction

Respiratory syncytial virus (RSV) induces robust CD8 T cell responses that play a critical role in controlling virus replication and determining the progression of disease in animal models and infected humans. For example, autopsies of children with fatal RSV infections show a relative deficiency of CD8 T cell responses[1]; recipients of allogeneic bone marrow and lung transplants have difficulty in controlling RSV replication and often have fatal outcomes as a consequence of syncytium-forming pneumonia[2, 3]; and in patients with severe combined immunodeficiency (SCID), RSV infection results in persistent virus shedding that can be controlled with T cell reconstitution[4, 5]. Adoptive transfer of effector CD8 T cells can clear persistent RSV shedding in immunodeficient mice[6]; and depletion of T cells in mice results in persistence of the virus[7, 8]. However, the effect of CD8 T cell responses is not always beneficial. While T cell reconstitution reduces RSV load in SCID patients, it causes significant pulmonary inflammation[5]. In mice, passive transfer of a large amount of CD8 T cells in the setting of RSV infection results in hemorrhagic pneumonia[6], while depletion of CD8 T cells reduces disease severity[7]. Achieving an acceptable balance between protective immunity and immunopathology has been an elusive goal for RSV vaccine development and is a key objective for programs developing preventive and therapeutic strategies for pathogens requiring T cell-mediated immunity.

CD8 T cells are a heterogeneous population with phenotypically and functionally diverse subsets, and viral infection often induces a broad spectrum of CD8 T cell responses[9, 10]. Most studies report bulk CD8 T cell responses, or are focused on CD8 T cells targeting a relatively small number of epitopes. Those immunodominant epitopes are often discovered and selected as endpoints in evaluation of vaccination and immune intervention. Quantitative assessment of bulk CD8 T cell responses has shown little correlation with control of virus replication, and numerically subdominant T-cell responses have been demonstrated to play important roles in immunity against selected viral infections[11, 12], particularly in settings where multiple epitopes are recognized or where immune escape mutations are common[13]. Therefore, it is important to characterize CD8 T cell responses not only by magnitude, but also by functional properties, breadth, location, durability, and contribution to viral clearance and immunopathology.

We took advantage of the CB6F1 hybrid mouse model to characterize CD8 T cell subsets and their role in response to RSV infection. Although semi-permissive, mice infected by intranasal inoculation of RSV generate robust T cell and antibody responses, and pulmonary inflammation. The kinetics of virus replication and progression of the disease is highly reproducible[14–16]. In CB6F1 mice, the dominant CD8 T cell responses of each parent strain assume a distinct hierarchy, in which response to H2-D^b^-restricted M_187-195_ epitope (D^b^M_187_) is numerically subdominant to response to the H2-K^d^-restricted M2_82-90_ epitope (K^d^M2_82_)[17]. The numerically dominant K^d^M2_82_ T cell response lyses RSV-infected cells and reduces viral load, but is also associated with infection-induced immunopathology and severe illness[18, 19]. Mutating this epitope leads to reduced disease and pathology[20]. This observation suggested that this high magnitude and easily measurable response may do more harm than good, and that other subdominant responses could effectively clear virus with less immunopathology. Here, we defined the attributes of the numerically subdominant D^b^M_187_ T cell response that targets the RSV matrix protein. We compared the cytotoxicity and control of virus replication, as well as the associated lung pathology and illness to that associated with K^d^M2_82_ T cells to help define properties that should be targeted by vaccines designed to elicit immunity mediated by CD8 T cells.

## Results

### RSV-specific CD8 T cell responses have epitope-dependent expansion capacity

**Fig 1:**
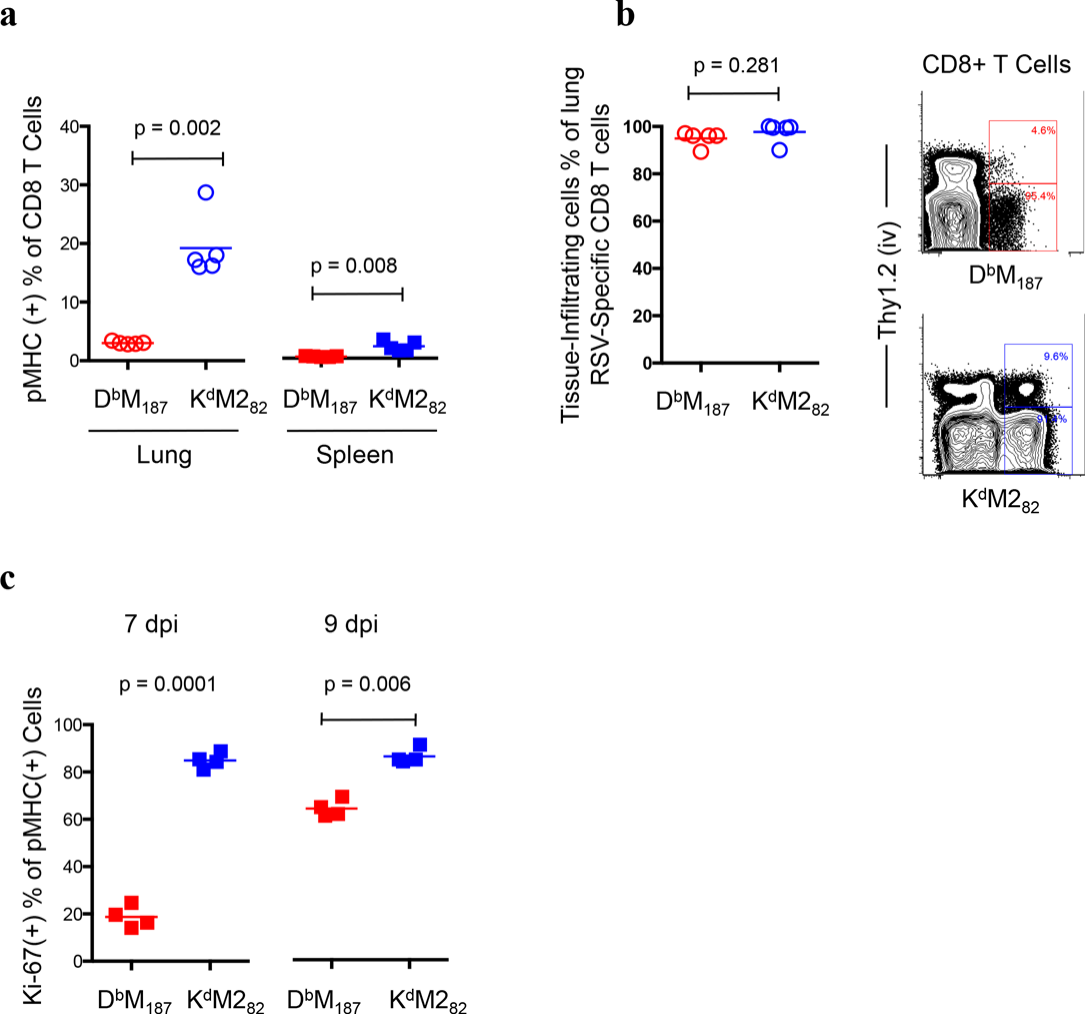
Numerical dominance of K^d^M2_82_ T cell response is associated with expansion capacity. (**a**) Magnitude of D^b^M_187_ and K^d^M2_82_ T cell response at 7 dpi. Frequency of D^b^M_187_ and K^d^M2_82_ T cells in CD8 T cell population were quantitatively assessed with flow cytometry. Data represent 5 independent experiments (n = 5/group/experiment). **(b**) The D^b^M_187_ and K^d^M2_82_ T cells infiltrate into lung parenchyma following RSV infection. Lung lymphocytes were isolated from RSV- infected mice at 7 dpi. T cells in vasculature were pre-labeled by intravenous anti-Thy1.2 staining prior to euthanizing mice. Proportion of D^b^M_187_ and K^d^M2_82_ T cells in lung parenchyma were quantitatively assessed with flow cytometry. Data represent 3 independent experiments (n = 5/group/experiment). (**c**) Lymphocytes isolated from spleens at 7 and 9 dpi were studied for Ki-67 expression by flow cytometry. The frequencies of Ki-67(+) cells in D^b^M_187_ and K^d^M2_82_ T cell subsets are shown. Data represent 3 independent experiments (n = 4/group/experiment). All data are shown as mean with independent data point and compared by Student t-test. Each symbol represents one mouse.

Following RSV infection, expanded D^b^M_187_ and K^d^M2_82_ T cells were quantified by fluorochrome-conjugated epitope peptide-MHC class I complexes (pMHCs) with flow cytometry (**S1 Fig**). We previously reported that D^b^M_187_ and K^d^M2_82_ T cells assumed a numerical hierarchy in lung[17], the primary site of RSV infection. Here, we show a similar hierarchy in spleen at relatively low magnitude (**Fig 1A**, p < 0.001 and p = 0.001, D^b^M_187_ and K^d^M2_82_ T cells respectively). Using a well-developed *in vivo* labeling procedure to distinguish cells in lung parenchyma from those in vasculature[21, 22], we found both D^b^M_187_ and K^d^M2_82_ T cells preferentially infiltrated into lung tissue (**Fig 1B**). Since these two subsets have similar precursor frequency in naïve mice and similar infiltrating capacity into the inflammatory site, there are other factors that determine the hierarchy[23]. The Ki-67 expression showed that K^d^M2_82_ T cells were more proliferative than D^b^M_187_ T cells (**Fig 1C**), consistent with the numerical dominance.

### D^b^M_187_ T cells have superior cytolytic activity

**Fig 2:**
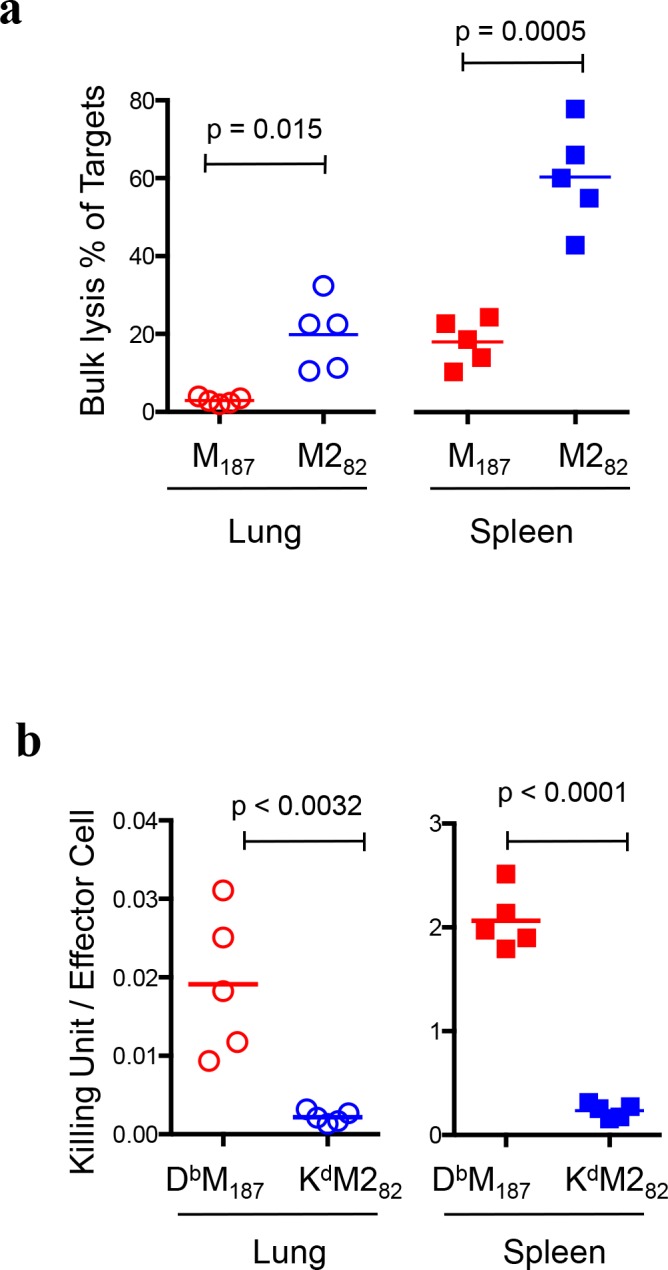
K^d^M2_82_ T cells dominate bulk cytotoxicity but D^b^M_187_ T cells have superior individual cytotoxicity. (**a)** Bulk cytotoxicity of D^b^M_187_ and K^d^M2_82_ T cells *in vivo*. The epitope peptide-loaded and fluorochrome-labeled targets, as well as OVA_257_ peptide-loaded and fluorochrome-labeled controls, were co-transferred into RSV-infected mice at 7dpi, and recovered 3 hours later. The recovery ratio were assessed with flow cytometry and compared with recovery ratio from naïve recipients to calculate epitope-specific lysis. Data represent 5 independent experiments (n = 5/group/experiment). **(b)** Cytotoxicity of individual D^b^M_187_ and K^d^M2_82_ T cells. Ratio of specific lysis of donor targets in (a) was divided by frequency of endogenous D^b^M_187_ and K^d^M2_82_ T cells respectively to quantitatively express arbitrary “Killing Unit” of individual cells. Data represent 5 independent experiments (n = 5/group/experiment). All data are shown as mean with independent data point and compared by Student t-test. Each symbol represents one mouse.

To functionally evaluate D^b^M_187_ and K^d^M2_82_ T cell cytotoxicity *in vivo*, we co-transferred M_187_ or M2_82_ peptide-loaded naïve spleen cells as targets with OVA_257_ peptide-loaded controls into RSV-infected and naïve recipients, and recovered the donor cells 3 hours later. The donor target and control cells were identified and distinguished by fluorochrome spectra and fluorescence intensity (**S2 Fig**). Recovery ratio of live targets to controls from the infected recipients was compared to recovery ratio from naïve recipients to calculate specific cytotoxicity[24]. We found that the bulk cytotoxic hierarchy correlated with overall numerical hierarchy, as more M2_82_ peptide-loaded targets were lysed in RSV-infected recipients than M_187_ peptide-loaded targets (**Fig 2A**). Although lung had higher D^b^M_187_ and K^d^M2_82_ T cell frequency and counts than spleen (**Fig 1A and S3 Fig**), there were more targets lysed in spleen than in lung (p = 0.0047 and p = 0.0016, M_187_ and M2_82_ peptide–loaded respectively). The data suggest that cytotoxic potency may differ at the individual effector cell level.

To evaluate individual cell cytotoxicity, we divided lysed ratio of donor targets by frequency of endogenous CD8 T effector cells with relevant pMHC specificity, and expressed cytotoxic potency of individual effector cells in “killing” units. This value represents a relative frequency of targets lysed by individual CD8 T effector cells. We found that individual D^b^M_187_ T cells lysed more targets than K^d^M2_82_ T cells (**Fig 2B**), and both subsets more efficiently lysed targets in spleen than they did in lung (p < 0.0001 for both D^b^M_187_ and K^d^M2_82_ T cells).

### D^b^M_187_ T cells have high avidity TCR and enhanced effector-function signaling pathways

**Fig 3:**
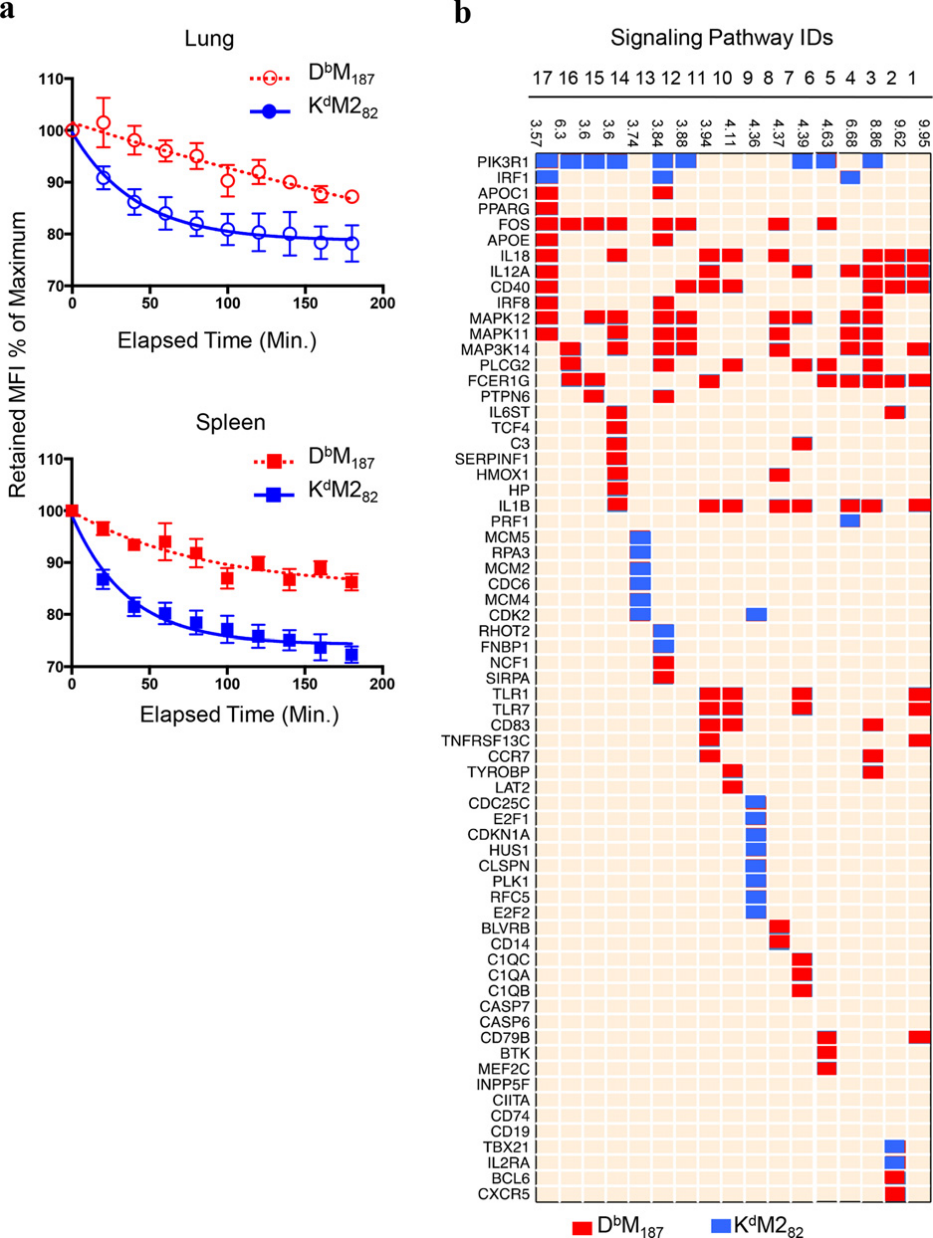
The D^b^M_187_ T cells express high avidity TCR and signaling pathways promoting cytotoxic function. **(a)** The TCR avidity was assessed by dissociation of D^b^M_187_ and K^d^M2_82_ from CD8 T cells. CD8 T cells were labeled with pMHCs and assessed cell-bound median fluorescence intensity (MFI) at indicated time point by flow cytometry. The MFI at 0 min was defined as the maximum measurement (100%). Data were analyzed with one-phase exponential decay using nonlinear regression, and shown at mean ± SEM of three independent experiments (n = 5/group/experiment). **(b)** Transcriptional expression of genes that are up-regulated after RSV infection and associated with conventional signaling pathways. The D^b^M_187_, K^d^M2_82,_ and bulk CD8 T cells were sorted from spleen lymphocytes by FACS at 7 dpi. The mRNAs were isolated, amplified and labeled, then hybridized onto Illumina Mouse Chips. The quantitative gene expression were analyzed and normalized. Genes with Log_2_ Fold Change (FC) > 1.3, p < 0.05 and FDR < 0.25 (listed on left side of the chat) and associated signaling pathways were shown (Pathways 1: Altered T Cell and B Cell Signaling in Rheumatoid Arthritis; 2: T Helper Cell Differentiation; 3: Dendritic Cell Maturation; 4: Type I Diabetes Mellitus Signaling; 5: Roe of NFAT in Regulation of the Immune Response; 6: Role of Pattern Recognition Receptors in Recognition of Bacteria and Viruses; 7: IL-10 Signaling; 8: Role of CHK Proteins in Cell Cycle Checkpoint Control; 9: TREM1 Signaling; 10: Communication between Innate and Adaptive Immune Cells; 11: CD40 Signaling; 12: Production of Nitric Oxide and Reactive Oxygen Species in Macrophages; 13: Cell Cycle Control of Chromosomal Replication; 14: Acute Phase Response Signaling; 15: CD28 Signaling in T Helper Cells; 16: PKC Signaling in T Lymphocytes; 17: IL-12 Signaling and Production in Macrophages.). Data were pooled from 10 or 11 individual mice in each group.

Since T cell cytotoxic potency has been associated with TCR avidity, we studied dissociation of pMHCs from cells as a surrogate measurement of TCR avidity. By measuring half-life (T_*1/2*_) of maximum median fluorescence intensity (MFI), we found that D^b^M_187_ tetramers were retained on CD8 T cells longer (59.62 and 405.7 min., lung and spleen respectively) than K^d^M2_82_ tetramers (25.07 and 28.51 min., lung and spleen respectively) (**Fig 3A**). The dissociation rate (*k*) was calculated using Equation of Dissociation Kinetics in Prism 6 (Version 6.0f), and demonstrated that D^b^M_187_ tetramer fell off CD8 T cells at lower rates (0.012 and 0.002, lung and spleen respectively) than K^d^M2_82_ tetramer (0.028 and 0.024, lung and spleen respectively). The data suggest that TCRs recognizing D^b^M_187_ have higher avidity than those recognizing K^d^M2_82_, and that avidity correlates with the individual cell cytotoxic potency. In addition, the data also indicate that CD8 T cells in spleen have higher TCR avidity than those in lung, suggesting that the binding capacity may be diminished in the setting of high antigen load and high level of T cell activation, or TCR may be down regulated.

Since TCR expression levels may have an effect on the expansion of antigen-specific cells, we assessed expression of TCR Vβ on CD8 T cells and found that K^d^M2_82_ T cells have slightly higher level of TCR expression than D^b^M_187_ T cells in lung, but not in spleen (**S4 Fig**). This difference may provide a partial explanation for the numerical hierarchy.

Transcriptional profiling of 8365 immune response-related genes revealed a distinct pattern in D^b^M_187_ and K^d^M2_82_ T cells (**S5A Fig**). Among the top 25 up-regulated genes in each subset, genes related to cytotoxic function and effector-memory differentiation (eg. *H2*, *cd40*, *and ccr6)* were up-regulated in D^b^M_187_ T cells, while genes related to cell division (eg. *cdk2 and cdc6*) were up-regulated in K^d^M2_82_ T cells (**S5B Fig**). Analyzing those with fold change (FC) ≥ Log_2_1.3, p < 0.05 and FDR < 0.25, the up-regulated gene sets in D^b^M_187_ T cells were related to effector-function pathways, such as cytotoxicity, cytokine and chemokine production, memory differentiation, co-stimulatory signaling and cell survival signaling, while those in K^d^M2_82_ T cells were related to promoting cell cycle and clonal expansion (**Fig 3B**). The expression of genes encoding cell-division cycle proteins, cell division cycle-associated proteins, mini-chromosome maintenance proteins, histones and cyclin-dependent kinases (**S5C Fig**) is consistent with post-transcriptional expression of Ki-67, which supports the findings that numerical hierarchy is associated with proliferative capacity.

### D^b^M_187_ T cells express an effector- and central-memory phenotype

**Fig 4:**
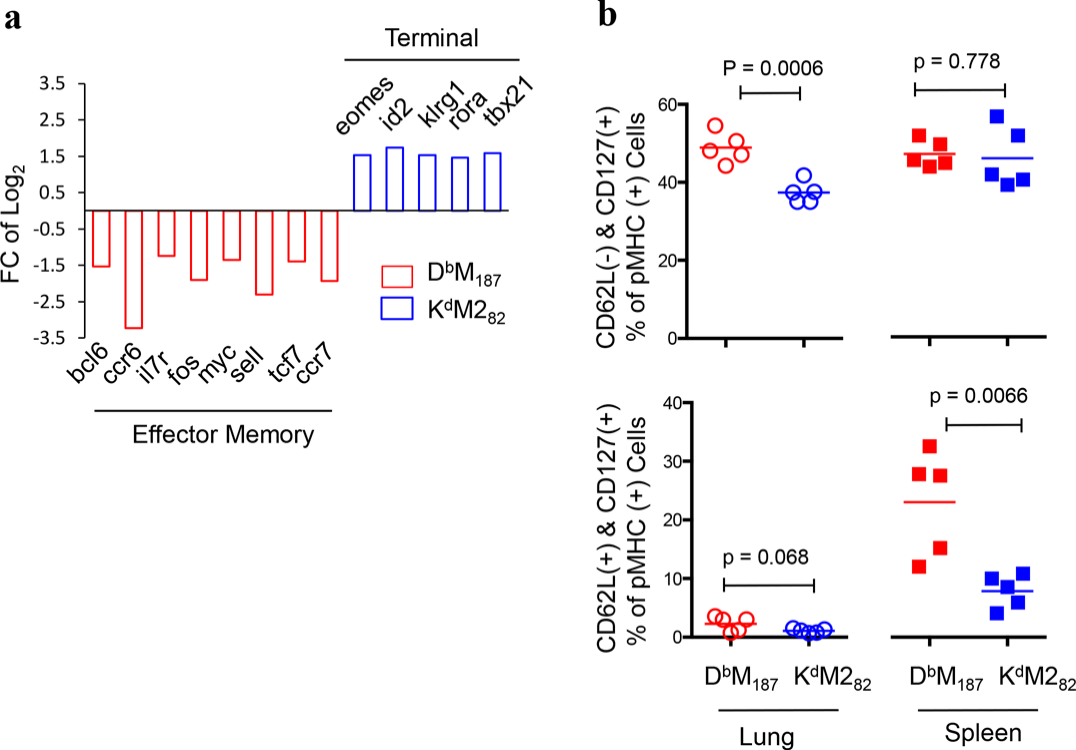
The D^b^M_187_ T cells differentiate toward to an effector- and central- memory phenotype. **(a)** Transcriptional expressions that are associated with cell differentiation were analyzed and normalized. Relative expression was calculated and presented as Log_2_ Fold Change (Log_2_FC). The Log_2_FC = Log_2_ K^d^M2_82_ –Log_2_ D^b^M_187_. Positive values indicate a specific gene expression was up-regulated in the K^d^M2_82_ subset, while negative values indicate up-regulated gene expression in the D^b^M_187_ subset. Genes with expression differences of FC > 1.3, p < 0.05 and FDR < 0.25 are listed. Data were pooled from 10 or 11 individual mice in each group. (**b**) Post-transcriptional expression of CD62L and CD127. The CD62L(-)CD127(+) and CD62L(+)CD127(+) frequencies at 7 dpi were assessed by flow cytometry and are shown as mean with independent data point and compared by Student’s *t*-test. Data represent 5 independent experiments (n = 5/group/experiment). Each symbol represents one mouse.

Comparing gene expressions related to cell differentiation, D^b^M_187_ T cells had elevated expression of *bcl6*, *ccr6*, *ccr7*, *il7r*, *fos*, *myc*, *sell* and *tcf7* (**Fig 4A**) that have been associated with effector-memory differentiation[25, 26]. In contrast, K^d^M2_82_ T cells had elevated expression of *eomes*, *id-2*, *klrg-1*, *roar* and *tbx21*, factors associated with terminal differentiation[25, 27, 28]. Phenotyping by flow cytometry confirmed the transcriptional profiles, in that more than 50% of D^b^M_187_ T cells were CD62L(-) and CD127(+), a phenotype of effector memory, compared to 37% of K^d^M2_82_ T cells in lung, and 23% of D^b^M_187_ T cells were CD62L(+) and CD127(+), a phenotype of central-memory, in spleen, while only 9% of K^d^M2_82_ T cells expressed both memory markers (**Fig 4B** and **S6A Fig**). We assessed epitope-specific CD8 T cell populations and their phenotype at 8 weeks post RSV infection. Our data showed that D^b^M_187_ T cells have a higher frequency of cells with central memory phenotype (CD62L+ & CD127+) than K^d^M2_82_ T cells, and the central memory cell counts are the same although K^d^M2_82_ T cell have a greater total cell count denominator than D^b^M_187_ T cells at peak of response (**S6b Fig**). These data provide additional evidence that D^b^M_187_ T cells are more likely to differentiate towards a central memory phenotype and survive after virus clearance.

### Activated K^d^M2_82_ T cells are susceptible to inhibitory regulation and apoptosis

**Fig 5:**
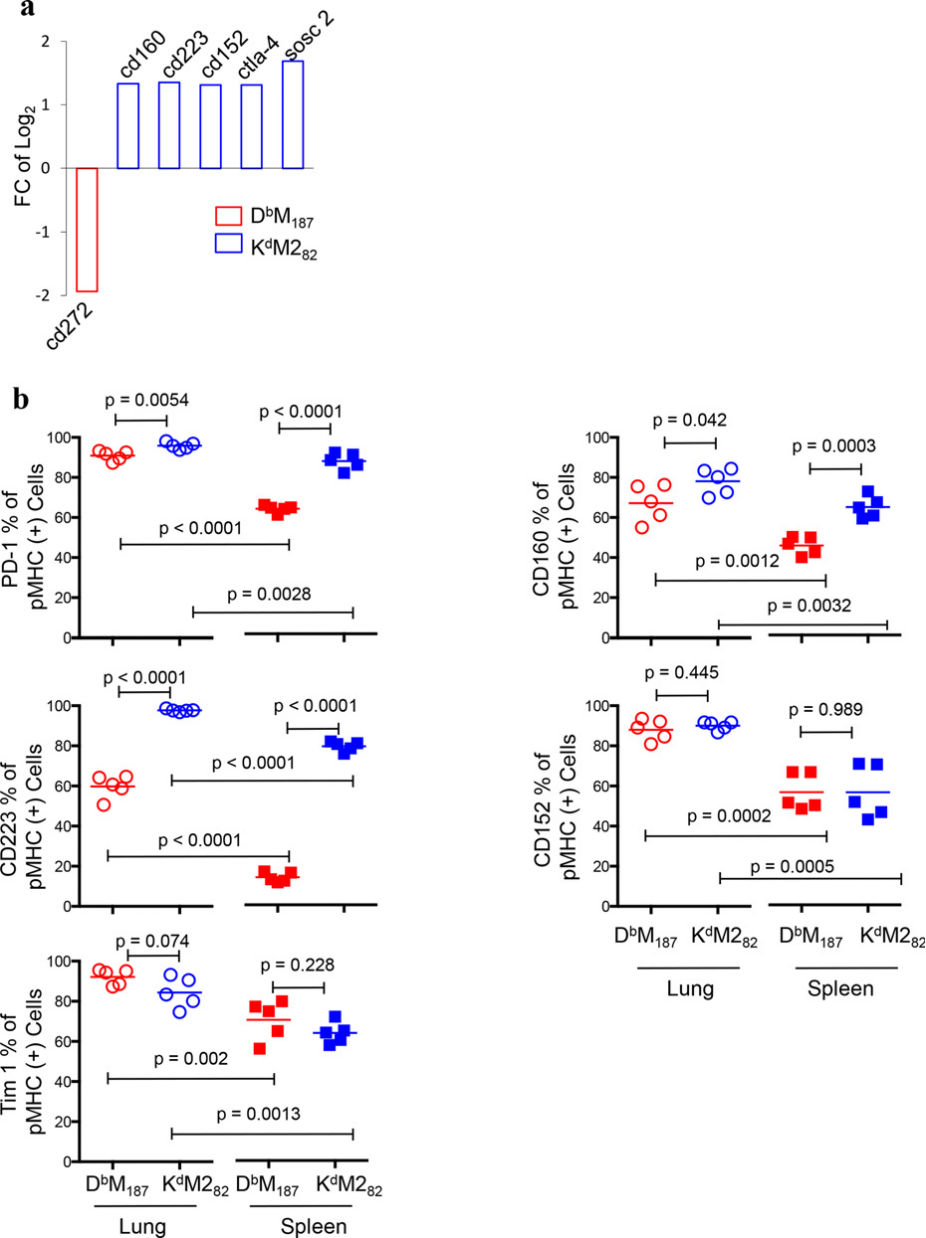
Activated K^d^M2_82_ T cells up-regulate expression of inhibitory receptors. **(a)** Transcriptional expression of genes encoding inhibitory receptors with FC > ±1.3, p < 0.05 and FDR < 0.25 were listed. Data were pooled from 10 or 11 individual mice in each group. (**b**) Post-transcriptional expression of inhibitory receptors at 7 dpi was assessed by flow cytometry. The frequencies are shown as mean with independent data point and compared by Student’s *t*-test. Data represent 5 independent experiments (n = 5/group/experiment). Each symbol represents one mouse.

T cell function is regulated by inhibitory receptors. Transcriptional profiling revealed that K^d^M2_82_ T cells up-regulated expression of multiple inhibitory receptor genes (**Fig 5A**), such as *cd160*, *cd223*, *cd152*, *ctla-4* and *sosc2*[29]. This is consistent with our phenotype study that showed PD-1, CD160 and CD223 expression was more common on K^d^M2_82_ T cells than on D^b^M_187_ T cells (**Fig 5B**). Although D^b^M_187_ T cells elevated *cd272* (*btla*) expression, another inhibitory receptor gene, the post-transcription level was similar to that of K^d^M2_82_ T cells (**S7A Fig**). We also found that cells isolated from lung were more likely to express inhibitory receptors than those isolated from spleen, including CD152, Tim1 and Tim3, although the difference in Tim3 expression was not statistically significant (**S7B Fig**). The transcriptional profile and phenotype of inhibitory receptors inversely correlate with individual D^b^M_187_ and K^d^M2_82_ T cell cytotoxic potency and suggest that the K^d^M2_82_ T cells are more susceptible to inhibitory regulation than the D^b^M_187_ T cells.

**Fig 6:**
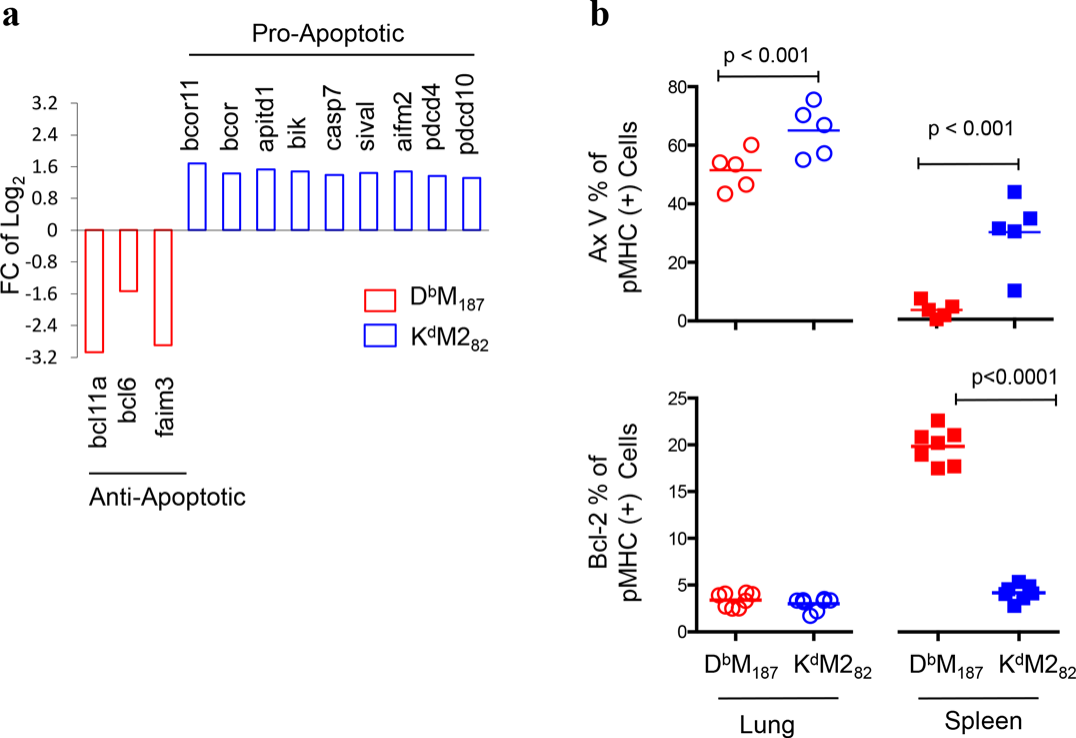
Activated K^d^M2_82_ T cells are apoptotic. **(a)** Transcriptional expression of genes encoding pro- and anti-apoptosis molecules with FC > ±1.3, p < 0.05 and FDR < 0.25 were listed. Data were pooled from 10 or 11 individual mice in each group. (**b**) Apoptotic cells were identified by Annexin V staining, and post-transcriptional expression of Bcl-2 was identified by monoclonal antibody with flow cytometry at 7 dpi. The frequencies are shown as mean with independent data point and compared by Student’s *t*-test. Data represent 3 or 4 independent experiments (n = 5/group/experiment). Each symbol represents one mouse.

TCR ligation triggers activation-induced cell death that also negatively regulates T cell function. The expression of pro-apoptotic genes, such as *bcor*, *bcorl1*, *apitd1*, *bik*, *casp7*, *sival*, *aimf2*, *pdcd4* and *pdcd10*[30–38], were up-regulated in K^d^M2_82_ T cells, while those associated with anti-apoptosis, such as *bcl11a*, *bcl6* and *faim3[39–41]*, were up-regulated in D^b^M_187_ T cells (**Fig 6A**). The *bcl11a* was also one of the top 25 up-regulated genes in D^b^M_187_ T cells (**S5B Fig**). Flow cytometry analysis showed that K^d^M2_82_ T cells were more likely to be stained with Annexin V than D^b^M_187_ T cells, which is an early indication of apoptotic change (**Fig 6B)**. In contrast, D^b^M_187_ T cells were more likely to express Bcl-2 than K^d^M2_82_ T cells. Both CD8 T cell subsets from lung were more apoptotic than those from spleen (p < 0.0001, D^b^M_187_ and K^d^M2_82_ T cells). The pattern of pro- and anti-apoptotic molecule expression at both transcriptional and protein levels suggests that a large proportion of the CD8 T cells, particularly K^d^M2_82_ T cells, progress towards apoptosis at the site of inflammation.

### D^b^M_187_ T cells efficiently control viral replication with limited pulmonary inflammation and disease severity

**Fig 7:**
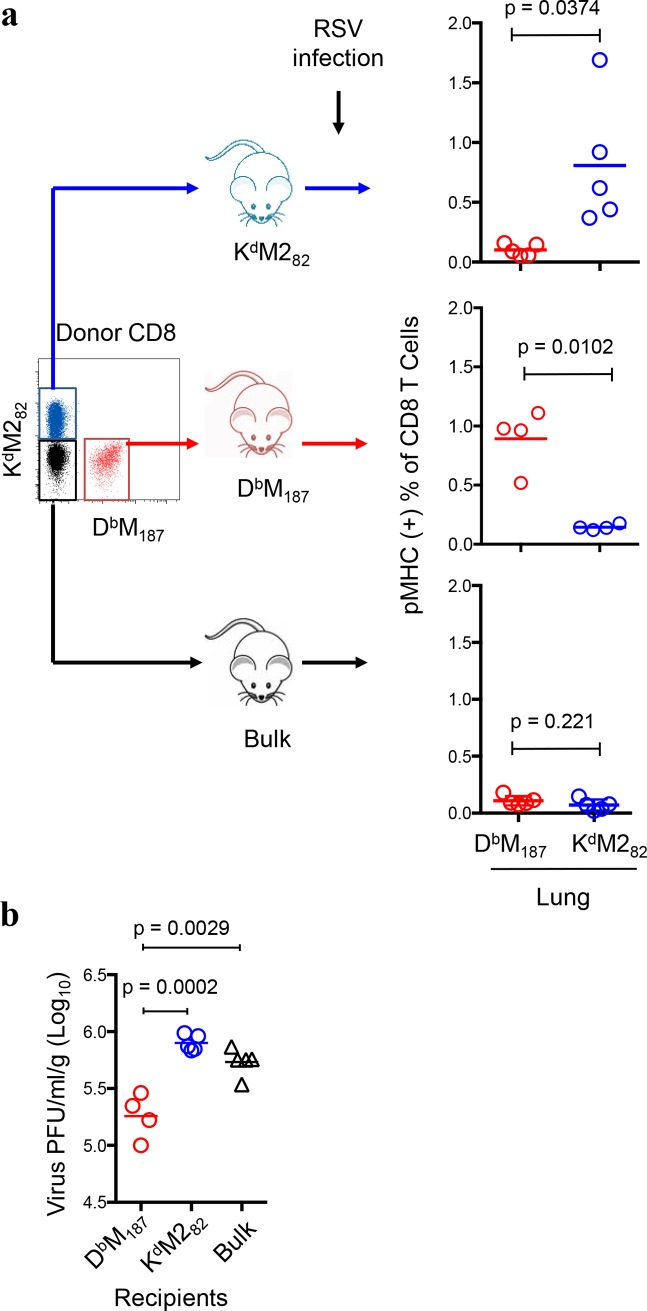
The D^b^M_187_ T cells efficiently control viral replication. **(a)** Adoptive transfer of pMHC-specific donor cells increases precursor of CD8 T effector cells during early infection. Live D^b^M_187_, K^d^M2_82_ and bulk (with neither specificity) CD8 T cells from spleen lymphocytes of RSV-infected mice at 7dpi were sorted with FACS, and transferred into naive recipients respectively. The recipients were then challenged with RSV next day, and were evaluated for the donor D^b^M_187_ and K^d^M2_82_ T cell frequencies in the right lung at 4 dpi by flow cytometry. **(b)** Viral activity in RSV challenged recipients. Left lungs of the RSV-challenged recipients were assessed for virus replication. The virus titers are expressed as log_10_ PFU/gram of lung tissue. Data are shown as mean with independent data point and compared by Student’s *t*-test. Data represent 3 independent experiments (n = 4 or 5/group/experiment). Each symbol represents one mouse.

To evaluate capacity of controlling virus replication *in vivo*, we sorted live D^b^M_187_, K^d^M2_82_, and bulk (with neither specificity) CD8 T cells by FACS from the spleen of RSV-infected mice and transferred equal numbers into naive recipients. The recipients were then challenged with RSV one day later. At 4 days post infection (4 dpi), we detected 0.9 ± 0.3% D^b^M_187_ and 0.8 ± 0.5% K^d^M2_82_ T cells (mean ± SD) in recipient’s lung CD8 T cell population respectively. The epitope-specific CD8 T cells were not detectable above background (≤ 0.05% of CD8 T cells) in mice that received bulk CD8 T cells or CD8 T cells with irrelevant pMHC specificity (**Fig 7A**). This indicates that virtually all the epitope-specific CD8 T effector cells in lung at 4 dpi are donor origin. This is consistent with previous studies that have shown endogenous RSV-specific CD8 T cells are rarely detectable at 4 dpi of primary infection. Assessing anti-viral activity of isolated lung samples *in vitro*, we found that D^b^M_187_ T cell recipients had significantly less viral replication in lungs than K^d^M2_82_ or bulk CD8 T cell recipients (**Fig 7B**). K^d^M2_82_ T cell recipients had similar viral replication in lungs as bulk CD8 T cell recipients (p = 0.115). We assessed IFN-γ in lung tissue homogenates, and found no significant difference among the recipient groups (**S8 Fig**). Although D^b^M_187_ T cells have more efficient effector cytokine production at the individual cell level than K^d^M2_82_ T cells, the impact of cytokines from adoptively transferred donor cells is likely to be masked by high levels of endogenous cytokine production.

**Fig 8:**
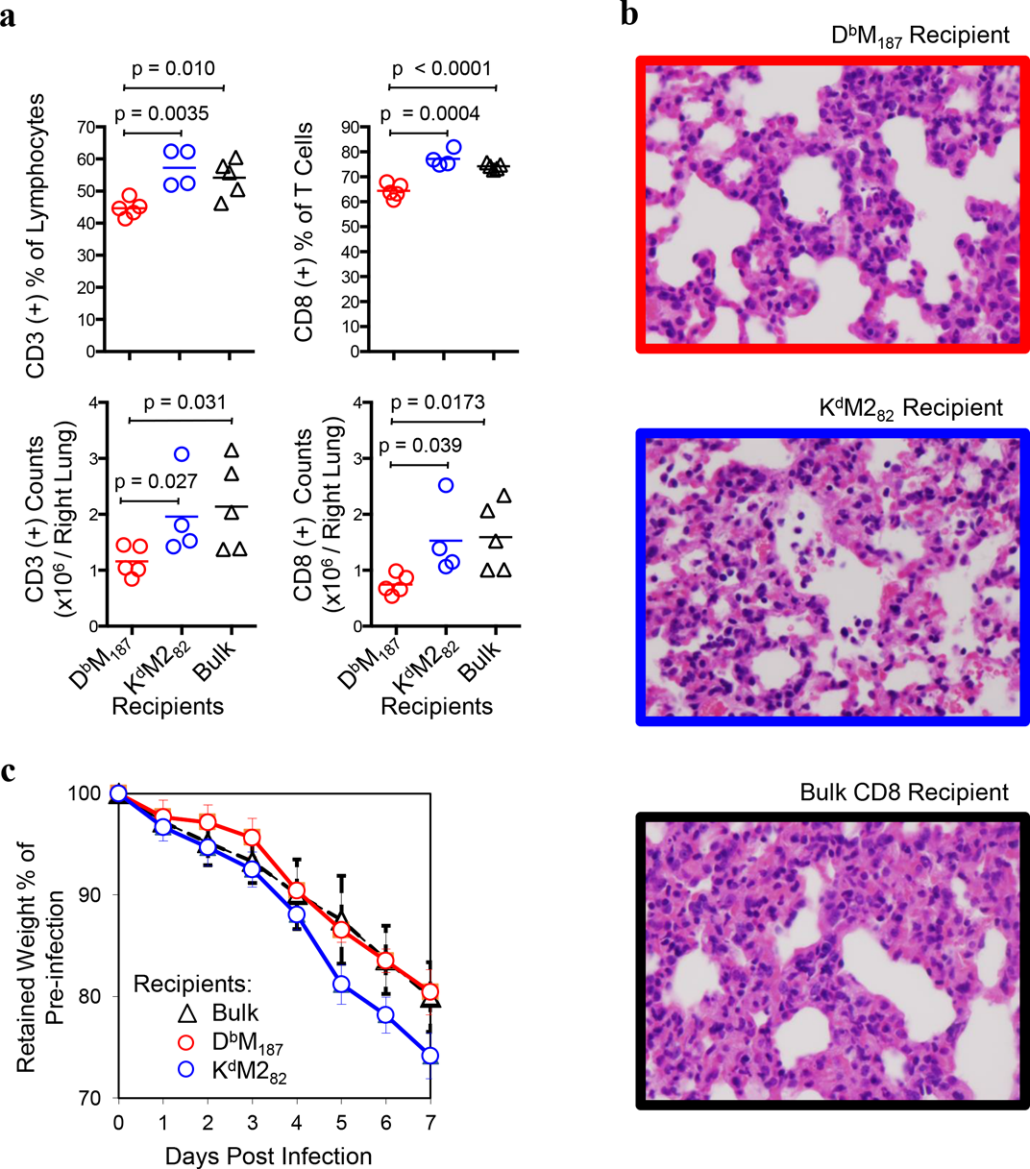
The D^b^M_187_ T cells provide protection with limited pulmonary inflammation and illness of the recipients. The D^b^M_187_, K^d^M2_82_ and bulk CD8 T cell recipients were challenged with RSV **(a)** Inflammatory cells were assessed at 7 dpi with flow cytometry. The absolute number and frequency of CD3 (+) cells and CD8 (+) T cells are expressed as mean with independent data point and are compared by Student’s *t*-test. Data represent 3 independent experiments (n = 5/group/experiment). Each symbol represents one mouse. (**b**) Pathology of lung. Left lung of the virus-challenged recipients were studied for histological change at 7 dpi. Microscopic study represents 3 independent experiments (n = 5/group/experiment). **(c)** Severity of illness. The virus-challenged recipients were weighed daily, from 0 to 7 dpi. Percentage represents retained proportion of pre-infection weight (mean ± SEM). Data represent 3 independent experiments (n = 5/group/experiment) and are compared by Student *t*-test.

Adoptively transferred donor cells increased recipient precursor frequency of relevant pMHC specificity and slightly altered the endogenous hierarchy at the day 7 peak response (**S9 Fig**). D^b^M_187_ T cell recipients had a reduced disparity of CD8 T cell responses (K^d^M2_82_: D^b^M_187_ = 3: 1) compared to bulk CD8 T cell recipients (K^d^M2_82_: D^b^M_187_ = 5:1), while the K^d^M2_82_ T cell recipients had exaggerated disparity (K^d^M2_82_: D^b^M_187_ = 10:1). Histopathological evaluation of lung tissue revealed D^b^M_187_ T cell recipients had decreased frequency and count of total T and CD8 T cells compared to bulk CD8 and K^d^M2_82_ T cell recipients (**Fig 8A**). The K^d^M2_82_ T cell recipients had similar T and CD8 T cell frequencies and total cell counts in lung compared to the bulk CD8 T cell recipients. There was no significant difference in CD4 T cell counts between groups (**S10 Fig**). Paraffin-embedded sections of formalin-inflated lungs at 7 dpi were stained with hematoxylin-eosin and evaluated by an independent pathologist in a blinded manner. Typically, pathology after primary infection is most severe between 8 and 10 dpi. The histopathology was analyzed on 7 dpi because the transfer of immune competent cells accelerated the time course of response. Mice without RSV infection had few infiltrates with few mononuclear cells present in the interstitium (**S11 Fig**). After RSV infection, lung shows inflammatory histology. Although the lung pathology of recipient groups did not show significant difference by an over-all pathology score, 13.85±1.10, 12.28±0.79 and 15.43±1.21 for D^b^M_187,_ K^d^M2_82_ T cell and bulk CD8 T cell recipients, respectively (mean±SD in a 1–15 scale level), the K^d^M2_82_ T cell and bulk CD8 T cell recipients showed more peribronchiolar neutrophil infiltration and higher magnitude interstitial inflammation and macrophage infiltration, suggesting a greater non-specific inflammatory response compared to D^b^M_187_ T cell recipients. Bulk CD8 T cell recipients had an intermediate phenotype with interstitial infiltrates composed of both mononuclear cells and polymorphonuclear leukocytes (PMNs), but without frank alveolitis (**Fig 8B**). Recipients of the immunodominant K^d^M2_82_ T cells had hemorrhagic alveolitis characterized by inflammatory cells and red blood cells (RBCs) frequently found in the alveolar space. The interstitial and alveolar infiltrates were composed primarily of mononuclear cells, but also contained a relative high frequency of PMNs. There was also evidence of epithelial necrosis with more pyknotic nuclei in this group. This is similar to the original report of CD8 transfer in H-2^d^ BALB/c mice causing hemorrhagic alveolitis[6]. The D^b^M_187_ T cell recipients had mononuclear cell interstitial infiltrates with rare PMNs and no cells in the alveolar space. This study indicates that the K^d^M2_82_ T cells cause more tissue destruction and a more severe immunopathology than D^b^M_187_ T cells. We also monitored body weight change after virus challenge as a surrogate for severity of illness. All mice lost weight after RSV infection. However, the K^d^M2_82_ T cell recipients had earlier and greater weight loss than other two groups. The differences were statistically significant from 5 to 7 dpi (**Fig 8C**, p < 0.05–0.005). The virus clearance, pathology and weight loss data indicate that D^b^M_187_ T cell-mediated virus clearance is more efficient and less pathogenic than that mediated by K^d^M2_82_ T cells.

## Discussion

It has become increasingly evident that magnitude of CD8 T cell response does not necessarily correlate with the extent of protection from infectious pathogens, and that functional properties of T cells may be more predictive of infection outcome[11, 12, 42]. In addition, the analysis of polyclonal T cell responses in bulk masks the functional diversity of T cells responding to specific epitopes. Even within an epitope-specific response, there are clonotype-specific subsets, each with their own functional profiles that collectively determine the characteristics of the overall response[9, 10]. Defining the characteristics of CD8 T cells that can efficiently clear virus-infected cells with minimal immunopathology has obvious implications for developing interventions that provide T cell-mediated immunity.

In the CB6F1 hybrid mouse model of RSV infection, numerically dominant K^d^M2_82_ T cell response account for the majority of bulk cytolytic activity. But individual K^d^M2_82_ T cells are relatively deficient in effector functions, and only about half of them produce IFNγ, which is a key molecule in eliminating virus infected cells[17, 43, 44]. Dramatically increasing K^d^M2_82_ T cells to control RSV replication results in elevated pulmonary inflammation and disease severity[17, 18]. Informed by an earlier finding that virus clearance could be accomplished in mice infected with virus containing a mutated M2_82_ T cell epitope[20], we evaluated numerically subdominant CD8 T cell responses and found that individual D^b^M_187_ T cells were more efficient at lysing infected cells and controlling virus replication. CD8 T effector cells pre-existing in mucosal tissue have been reported to eliminate infection at the point of initial virus replication[45]. Adoptive transfer of D^b^M_187_ T cells increased pre-existing pulmonary CD8 T effector cells and controlled the virus replication during early infection. Prompt control of virus replication resulted in a mild infection-induced lung inflammation, with fewer infiltrating T cells, mononuclear cells and PMNs, and no hemorrhagic alveolitis or epithelial necrosis displayed in histological sections. As a consequence of the limited damage to lung tissue and function, the D^b^M_187_ T cell-mediated protection resulted in less illness than K^d^M2_82_ T cell-mediated viral clearance. The efficiency of protection against virus challenge is an intrinsic property of D^b^M_187_ T cell cytolytic function, and is not associated with differences in migration, since K^d^M2_82_ T cells have similar or higher presence in host lung[46].

High TCR avidity has been suggested as a critical factor for induction of CD8 T cell effector function in viral infections[47, 48]. Our previous studies revealed that D^b^M_187_-specific TCRs recognize a more complex epitope structure and tend to have private clonotypes, presumably with a greater surface area of interaction with the epitope than K^d^M2_82_-specific TCRs[49]. Here, our transcriptional profiling showed that the engagement of the high avidity D^b^M_187_-specific TCR activated pathways to promote effector function, survival, and effector-memory differentiation. The association of cytolytic potency with enhanced co-stimulation signaling, cytokine and chemokine production has been well studied and demonstrated to be a critical factor in anti-viral immunity[50]. The effector-memory phenotype is a subpopulation of CD8 T cells with particularly good functional activity. For example, CD8 T cells expressing an effector-memory phenotype were shown to be critical for protection from mucosal SIV challenge[51] and could clear virus-infected cells at an early stage of infection[52]. Our transcriptional, post-transcriptional, and long-term survival data suggest that D^b^M_187_-specific TCR engagement elicits a program driving cells toward effector-memory differentiation with higher cytotoxic potency than terminally differentiated cells driven by K^d^M2_82_-specific TCR engagement.

Replication capacity accounts for the numerical dominance of K^d^M2_82_ T cell response. In some studies, precursors determine the magnitude of response[53, 54]. Our early study has showed that the frequency of D^b^M_187_ and K^d^M2_82_ T cells are about the same in naïve mice[23]. Here, we showed that moderately increasing precursors of the subdominant subset by adoptive transfer influenced but did not reverse the hierarchy pattern. Difference in TCR level may provide partial explanation to the formation of numerical hierarchy but is not likely to be the major determinant. Recent studies reported that the level of TCR expression had minimal effect on the magnitude of antigen-specific CD8 T cells expansion[55, 56]. We also found that the differences in TCR expression levels were not proportional to differences in response magnitudes, and there is no significant difference in TCR expression levels on splenocytes. Variation in antigen dose may affect CD8 T cell expansion. But in our previous experiment using viral vector to express M and M2 antigens as a fusion protein, in which both epitopes were equally available and processed, we showed similar CD8 T cell response hierarchy as observed in RSV infection[23]. We propose that the extreme proliferation of K^d^M2_82_ T cells is presumably driven by activation signals derived from the pMHC-TCR interaction. K^d^M2_82_ T cells express TCRs that recognize a relatively flat and featureless epitope, and have a highly public clonotype[10]. These characteristics determine that K^d^M2_82_ T cells have distinct activation thresholds for proliferation and cytolytic activity or cytokine production. It has been reported that some epitope-specific CD8 T cells can be expanded by cognate peptide in the absence of helper cell-derived signals, although their effector functions might not be triggered[57]. Another study based on mathematic modeling concluded that rapid expanding T cells were likely to be deficient in effector function and short-lived[52]. It is possible that the rapidly dividing population has a diminished TCR avidity due to down-regulation. The type of exaggerated activation that leads to the numerical dominance of K^d^M2_82_ T cell response may be selected by immunogenicity testing based solely on magnitude of response, but is not the preferred adaptive immune response from the host perspective.

Our previous study reported that the K^d^M2_82_ T cells were selectively susceptible to regulatory CD4 T cells[20, 58]. Here, we showed that the K^d^M2_82_ T cells were subject to inhibitory signals, by elevated expression of PD-1, CD160, CD223 and other inhibitory receptors. These inhibitory receptors have a negative impact on T cell effector function and control of viral replications in chronic[29] and acute infections[59]. RSV infection has been reported to induce PD-L1 expression on bronchial epithelial cells, which resulted in an inhibition of CD8 T cell antiviral activity[60]. The PD-1 and other inhibitory signals can also suppressed memory formation and directed CD8 T cells to terminal differentiation[29]. Being apoptotic has been shown to negatively impact effector function. The extremely activated and robustly proliferating K^d^M2_82_ T cells up-regulate genes promoting apoptosis, but have reduced expression of genes encoding anti-apototic molecules. The inhibitory receptor expression and pro-apoptotic changes are consistent with the functional deficiency of K^d^M2_82_ T cells, and with previous observations that only a fraction of the K^d^M2_82_ T cells produce IFN-γ, while almost all D^b^M_187_ T cells are IFN-γ-producing[17, 44].

The tissue environment may also have impact on CD8 T cell effector function. Terminal differentiation and lack of cytolytic capacity have been previously demonstrated to be a characteristic of lung-derived CD8 T cells in response to viral infections[57]. Terminally differentiated CD8 T cells in the lung had limited cytolytic activity and were unable to control influenza and Sendai virus infection[61]. Here, we found that D^b^M_187_ and K^d^M2_82_ T cells isolated from lung, where they are needed to clear virus-infected cells, were less cytotoxic than cells isolated from spleen. Our data suggest that cells in the lung are vulnerable to suppressive regulation by elevating expression of inhibitory receptors, and susceptible to activation-induced cell death by down-regulating anti-apoptotic molecules. Terminal differentiation and loss of effector function has been associated with prolonged TCR ligation and inflammatory cytokine-driven differentiation[62–64]. Persistent engagement of TCR can trigger down-regulation of CD127 on CD8 T cells in lung[65], and adversely affect control of viral replication[66]. Thus, a balanced and modulated inflammatory response promotes optimal CD8 T effector function.

In the mouse model of RSV infection, a subdominant CD8 T cell response provided adequate control of viral replication during early infection, limited the viral replication, and reduced pulmonary inflammation and disease severity. These data support the concept that induction of T cells with high cytolytic activity are more favorable for protecting the host from viral infection than a high magnitude response with cells exhibiting a more extreme differentiation phenotype. The goal for vaccine-induced CD8 T cell-mediated protection should be to increase the precursor frequency of cells that can respond rapidly based on a pre-existing memory phenotype and localization. They should exhibit potent and efficient cytolytic activity without excessive proliferation that may lead to unwarranted immunopathology[12]. These findings raise the interesting possibility that by selecting for T cell epitopes with more complex structures, it may be possible to elicit T cell responses that have higher TCR-pMHC avidity. These cells would be more likely to achieve the activation threshold for initiating the effector functions needed to rapidly clear virus-infected cells with minimal immunopathology.

## Materials and Methods

### Mice and virus infection

Pathogen-free CB6F1 female mice between the ages of 8 and 10 weeks were purchased from Jackson Laboratories (Bar Harbor, ME) and cared for in accordance with the NIH Guide for the Care and Use of Laboratory Animals. The NIH Animal Care and Use Committee approved all animal protocols in this study. Experimental groups were age matched. RSV challenge stock was derived from the A2 strain by sonicating HEp-2 cell monolayers as previously described[16]. For infection, mice were inoculated with 1 x 10^7^ PFU live RSV in 10% EMEM intranasally under anesthesia with 3% isoflurane and oxygen.

### Ethics statement

Animal use and all procedures (VRC-13-443 and -447) were approved by the Vaccine Research Center, Animal Care and Use Committee (ACUC) in accordance with NIH policy and ARAC guidelines, and is accredited by the Association for Assessment and Accreditation of Laboratory Animal Care (AAALAC) and in compliance with the Animal Welfare Act and Public Health Service Policy on Humane Care and Use of Laboratory Animals. The National Institutes of Health Intramural Research Program OLAW assurance number is A4149-01.

### Cell culture and flow cytometry reagents

The cell culture medium was RPMI 1640 from HyClone (Logan, UT), supplemented with 10% heat inactivated fetal bovine serum, 2 mM glutamine, 10 U/ml penicillin and 10 μg/ml streptomycin. Fluorochrome-conjugated antibodies used to study cell phenotype, differentiation and other expressions with flow cytometry were anti-CD3–cyanine 7 (Cy7) allophycocyanin (APC), anti-Ki67-fluorescein isothiocyanate (FITC), anti-Bcl2-FITC, and Annexin V-FITC that were purchased from BD Biosciences (San Jose, CA). Unconjugated antibodies to CD4, CD8 and CD62L were purchased from Harlan (Indianapolis, IN) and were conjugated with Qdot605, Qdot655 and Alexa Fluor 688 from Invitrogen (Carlsbad, CA). The in-house conjugates were validated by comparison with commercial conjugates. Anti-PD-1-FITC, anti-CD272-phycoerythrin (PE), anti-CD152-PE, anti-Tim1-biotin, anti-Tim3-biotin, anti-CD223-PE and anti-CD127-Cy7PE were purchased from eBioscience Inc (San Diego, CA). Streptavidin (SA)-PE was purchased from ProZyme (Hayward, CA). Violet fluorescent reactive dye (Invitrogen, CA), anti-CD19- and anti-CD16/32-Pacific blue (eBioscience) were used as cell viability and lineage markers to exclude dead and non-T cells from analysis. Fluorochrome-conjugated epitope peptide–MHC class I complexes (pMHC), D^b^-M_187-195_-APC (D^b^M_187_-APC) and K^d^-M2_82-90_-PE (K^d^M2_82_-PE), were purchased from Beckman Coulter (Fullerton, CA).

### *In vivo* T cell labeling

Naïve or RSV-infected mice were injected intravenously with Cy5PE-conjugated anti-Thy1.2 antibody purchased from BioLegend (San Diego, CA). Mice were sacrificed 10 min. later. Lymphocytes from lung and spleen were isolated and stained for lineage differentiation and pMHC specificity following standard procedures, and then applied to flow cytometry analysis[21, 22].

### Cytotoxic activity assays

To evaluate cytotoxicity of D^b^M_187_ and K^d^M2_82_ T cells *in vivo*, we setup an assay based on previous described[24]. The targets were prepared from naïve spleen cells that were loaded with M_187-195_ (M_187_) or M2_82-95_ (M2_82_) peptides (Anaspec Inc, San Jose, CA) at a concentration of 10 μg/ml, then stained with 10 uM carboxyfluorescein succinimidyl ester (CFSE) or 10 uM CellTracker Red CMTPX (both from Invitrogen) respectively; controls were prepared from naïve spleen cells that were loaded with OVA_257-264_ (OVA_257_) peptide (Anaspec Inc) at a concentration of 10 μg/ml, then stained with either 0.1 uM CFSE or 0.2 uM CellTracker Red CMTPX respectively. These four populations, including M_187_-CFSE^high^, M2_82_-CMTPX^high^, OVA_257_-CFSE^low^ and OVA_257_-CMTPX^low^, each at 2 x 10^6^, were co-transferred into RSV-infected or naïve mice via tail vein. Cells from the lung and spleen were collected 3 hours later from the recipients. Donor cells were identified by fluorochromes spectra and distinguished by fluorescence intensity. The specific lysis were calculated using formula: ratio = (percentage fluorochrome^low^/percentage fluorochrome^high^). Specific lysis (%) = [1 − (ratio naive/ratio infected) × 100]. Frequency of pMHC-specific CD8 T cells was assessed simultaneously in the same cell preparation recovered. The cytolytic unit per pMHC specific CD8 T effector cell was calculated by dividing the percent of specific lysis by the frequency of pMHC-specific CD8 T cells.

### TCR avidity assessment

The pMHC decay assessment was performed as described previously[67]. Briefly, lung and spleen lymphocytes from RSV-infected mice were stained for lineage differentiation and epitope specificity with fluorochrome-conjugated pMHCs. Cells were washed and then incubated in the presence of anti-H2-D^b^ (12.5 μg/ml) or anti-H2-K^d^ (2.5 μg/ml) respectively, in culture medium containing 0.1% sodium azide at room temperature. Cells were recovered every 20 minutes, washed and then fixed with 1% paraformaldehyde and applied for flow cytometry to assess the intensity of fluorescence.

### Sorting and adoptive transfer of pMHC-specific donor cells

Live D^b^M_187_, K^d^M2_82_ and bulk (with neither specificity) CD8 T cells from spleen lymphocytes of RSV-infected mice at 7dpi were sorted by FACS. From each population, 5 x 10^5^ cells were transferred into naive recipients respectively via tail vein. The recipients were challenged with RSV 24 hours later, and were evaluated for the donor cell frequencies and the virus load.

### Viral load assessment

Lung tissue was sampled at indicated times after RSV infection and quickly frozen using ethanol-dry ice in 10% EMEM medium. After grinding and pelleting of tissue debris, serial dilutions of supernatant were inoculated onto 80% confluent HEp-2 cell monolayers in triplicate and overlaid with 0.75% methylcellulose in 10% EMEM. The cultures were incubated at 37°C for 4 days, then fixed with phosphate buffered 10% formalin and stained with hematoxylin and eosin. Plaques were counted and expressed as log_10_ PFU/gram of lung. The limit of detection is 1.8 log_10_ PFU/gram of lung.

### Histology

At indicated time points after RSV infection, mice were euthanized and the left lung was gently inflated with phosphate-buffered 10% formalin, then removed and placed in the buffered formalin. Thin sections were cut from paraffin-embedded lung tissues, stained with hematoxylin and eosin and examined by light microscopy.

### Microarray cDNA hybridization analysis

Splenocytes from RSV-infected mice were isolated and stained for lineage differentiation and pMHC specificities. D^b^M_187_ and K^d^M2_82_ T cells were sorted into RLT buffer (Qiagen, CA) with cell sorter Aria (BDbiosciences, CA). After isolation of mRNA, the total RNA was amplified and labeled using the Illumina TotalPrep RNA Amplification kit. The biotinylated cRNA was hybridized onto Illumina Mouse Chips and quantified using Illumina BeadStation 500GX scanner and Illumina BeadStudio v3 program. Illumina probe data were exported from BeadStudio as raw data and screened for quality. Samples failing chip visual inspection and control examination were removed. Gene expression data were analyzed using Bioconductor, an open-source software library for the analyses of genomic database on R, which is a language and environment for statistical computing and graphics (www.r-project.org). The R software package was used to first filter out probes with intensities below background in all samples, and then to replace minimum (a surrogate replacement policy) values below background using the mean background value of the built-in Illumina probe controls as an alternative to background subtraction (which may introduce negative values) to reduce “overinflated” expression ratios determined in subsequent steps, and finally quantitatively normalize the probe intensities. The resulting matrix showing probes as rows and samples as columns was log_2_ transformed and used as input for linear modeling using BioConductor Linear models for microarray analysis (LIMMA). The LIMMA package was used to identify differentially expressed genes (pFDR < 0.05).

### Accession numbers

Genes and proteins mentioned in the text can be found in the SwissProt data libraries (http://www.uniprot.org/uniprot/) under the following accession numbers: RSV-M (P03419), RSV-M2 (P04545), Ki-67 (Q61769), CD62L (P18337), CD127 (P16872), IFNγ (P01580), PD-1 (Q02242), CD160 (O88875), CD223 (Q61790), CD152 (P09793), Tim 1 (Q5QNS5), Annexin V (P48036), Bcl-2 (P10417).

## Supporting Information

S1 Fig Identification of D^b^M_187_ and K^d^M2_82_ T cells by flow cytometry. Lymphocytes isolated from the lung and spleen of RSV-infected mice at 7 dpi were stained for CD3, CD4 and CD8 lineage differentiation and pMHC-specificities. Dot plots represent strategies to identify D^b^M_187_ and K^d^M2_82_ T cells, by sequentially gating on singlets, lymphocytes, CD3(+) Dump(-), CD8(+) CD4(-), and pMHC(+) populations. The Dump channel included Violet fluorescent reactive dye, fluorochrom-conjugated anti-CD16/32 and anti-CD19 to exclude dead cells, monocytes, B cells, and other non-T cells. Dot plots represent 5 independent experiments (n = 5/group/experiment).
(TIF)

S2 Fig Quantitative assessment of peptide-loaded and fluorochrome-labeled targets and controls recovered from recipients in cytotoxicity assay *in vivo*. After adopting peptide-loaded and fluorochrome-labeled targets and controls, the recipient’s lung and spleen cells were isolated 3 hours later and analyzed by flow cytometry. The M_187_ and M2_82_ peptide-loaded targets were identified by high intensity of CFSE and CMTPX respectively; the OVA_257_ peptide-loaded controls were identified by lower intensity of CFSE and CMTPX and used as controls for the same fluorochrome-labeled targets respectively. Histograms show the proportion of cells with distinct fluorochrome labeling and intensity recovered from infected or naïve mice. Dot plots and histograms represent 5 independent experiments (n = 5/group/experiment).
(TIF)

S3 Fig The D^b^M_187_ and K^d^M2_82_ T cell counts in lung and spleen. The D^b^M_187_ and K^d^M2_82_ T cells from lung and spleen of infected mice at 7 dpi were quantitatively assessed by flow cytometry. Cell counts were calculated based on total cell counts in the lung and spleen cell preparations and the frequency of individual subsets. Data are shown as mean with independent data point and are compared by Student’s *t*-test. Data represent 5 independent experiments (n = 5/group/experiment). Each symbol represents one mouse.
(TIF)

S4 Fig TCR level of D^b^M_187_ and K^d^M2_82_ T cells. Lymphocytes isolated from the lung and spleen of RSV-infected mice at 7 dpi were stained for CD3, CD4 and CD8 lineage differentiation and pMHC-specificities. The expression of TCR on epitope-specific CD8 T cells was studied using fluorescence-conjugated anti-TCR Vβ (pan Vβ) monoclonal antibody, and the relative level of expression was measured by the medium fluorescence intensity (MFI). Data are shown as mean with independent data point and are compared by Student’s *t*-test. Data represent 2 independent experiments (n = 5/group/experiment). Each symbol represents one mouse.
(TIF)

S5 Fig Pattern of Transcriptional expressions of D^b^M_187_ and K^d^M2_82_ T cells. The D^b^M_187_, K^d^M2_82_ and bulk CD8 T cells were sorted from spleen by FACS at 7 dpi and studied for transcriptional expression of genes associated to immune responses. The quantitative gene expression were analyzed and normalized. Relative expression was calculated and presented as Log_2_ Fold Change (Log_2_FC). The Log_2_FC = Log_2_ K^d^M2_82_ –Log_2_ D^b^M_187_. Positive values indicate gene expression was up-regulated in the K^d^M2_82_ subset, while negative values indicate gene expression was up-regulated in the D^b^M_187_ subset. **(a)** All gene expressions with Log_2_ FC > ±1.3. **(b)** Top 25 up-regulated expressions among (a) in each subset. **(c)** Up-regulated expressions of genes in (a) related to MHC class I molecule and clone expansion. Each column represents data from one mouse.
(TIF)

S6 Fig Pattern of CD62L and CD127 expression and memory subsets. Lymphocytes were isolated from the lung and spleen of infected mice at 7dpi and studied for expression of CD62L and CD127 with flow cytometry. Dot plots show the gating strategy and represent 5 independent experiments (n = 5/group/experiment). **(b)** Lymphocytes were isolated from the lung and spleen of mice at 8 weeks post infection. The D^b^M_187_ and K^d^M2_82_ T cells were studied for their frequency, count, and expression of CD62L and CD127 with flow cytometry. Data are shown as individual data points with horizontal bar representing the mean, and are compared by Student’s *t*-test. Data represent 2 independent experiments (n = 5/group/experiment). Each symbol represents one mouse.
(TIF)

S7 Fig Similar expression of CD272 and Tim 3 in D^b^M_187_ and K^d^M2_82_ T cells. Lymphocytes were isolated from the lung and spleen of infected mice at 7dpi and studied for expression of (**a**) CD272 and (**b**) Tim 3 with flow cytometry. The frequencies are shown as mean with independent data point and compared by Student’s *t*-test. Data represent 5 independent experiments (n = 5/group/experiment). Each symbol represents one mouse.
(TIF)

S8 Fig Lung IFN-γ level of recipients after RSV challenge. Lung supernatant described in Fig 7B were collected and frozen saved. INF-γ level was assessed using LEGENDplex (BioLegend), a bead-based commercially available immunoassay service. The concentration was calculated referring to standard series. Each symbol represents one mouse (n = 11).
(TIF)

S9 Fig Adapting donor cells increases frequency of CD8 T cells with relevant specificity after virus challenge. After virus challenge, the recipients that adopted D^b^M_187_, K^d^M2_82_ and bulk CD8 T cells were evaluated for D^b^M_187_ and K^d^M2_82_ T cell frequencies in the right lung at 7 dpi with flow cytometry. The frequencies are shown as mean with independent data point and compared by Student’s *t*-test. Data represent 3 independent experiments (n = 4 or 5/group/experiment). Each symbol represents one mouse.
(TIF)

S10 Fig CD4 T cell counts were not significantly altered in recipients. The D^b^M_187_, K^d^M2_82_ and bulk CD8 T cell recipients were challenged with RSV. Inflammatory cells were assessed at 7 dpi with flow cytometry. The absolute number and frequency of CD4 (+) cells are expressed as mean with independent data point and are compared by Student’s *t*-test. Data represent 3 independent experiments (n = 5/group/experiment). Each symbol represents one mouse.
(TIF)

S11 Fig Histology of naïve lung. Left lung were isolated from naïve mice and studied for the histology of lung structure. Sections were examined under light microscope by independent blinded investigators. Histology study represents 3 independent experiments (n = 5).
(TIF)

## References

[1] Welliver TP, Garofalo RP, Hosakote Y, Hintz KH, Avendano L, Sanchez K, et al Severe human lower respiratory tract illness caused by respiratory syncytial virus and influenza virus is characterized by the absence of pulmonary cytotoxic lymphocyte responses. J Infect Dis. 2007;195(8):1126–36. Epub 2007/03/16. doi: JID37165 [pii] 10.1086/512615 . 17357048 PMC7109876

[2] Shah JN, Chemaly RF. Management of RSV infections in adult recipients of hematopoietic stem cell transplantation. Blood. 117(10):2755–63. Epub 2010/12/09. doi: blood-2010-08-263400 [pii] 10.1182/blood-2010-08-263400 . 21139081

[3] Milstone AP, Brumble LM, Barnes J, Estes W, Loyd JE, Pierson RN 3rd, et al A single-season prospective study of respiratory viral infections in lung transplant recipients. Eur Respir J. 2006;28(1):131–7. Epub 2006/03/03. doi: 09031936.06.00105505 [pii] 10.1183/09031936.06.00105505 . 16510454

[4] Hall CB, Powell KR, MacDonald NE, Gala CL, Menegus ME, Suffin SC, et al Respiratory syncytial viral infection in children with compromised immune function. N Engl J Med. 1986;315(2):77–81. Epub 1986/07/10. 10.1056/NEJM198607103150201 . 3724802

[5] El Saleeby CM, Suzich J, Conley ME, DeVincenzo JP. Quantitative effects of palivizumab and donor-derived T cells on chronic respiratory syncytial virus infection, lung disease, and fusion glycoprotein amino acid sequences in a patient before and after bone marrow transplantation. Clin Infect Dis. 2004;39(2):e17–20. Epub 2004/08/13. 10.1086/421779 CID32988 [pii]. . 15307047

[6] Cannon MJ, Openshaw PJ, Askonas BA. Cytotoxic T cells clear virus but augment lung pathology in mice infected with respiratory syncytial virus. J Exp Med. 1988;168(3):1163–8. Epub 1988/09/01. 3262705 10.1084/jem.168.3.1163 PMC2189034

[7] Graham BS, Bunton LA, Wright PF, Karzon DT. Role of T lymphocyte subsets in the pathogenesis of primary infection and rechallenge with respiratory syncytial virus in mice. J Clin Invest. 1991;88(3):1026–33. Epub 1991/09/01. 10.1172/JCI115362 1909350 PMC295511

[8] Tregoning JS, Yamaguchi Y, Harker J, Wang B, Openshaw PJ. The role of T cells in the enhancement of respiratory syncytial virus infection severity during adult reinfection of neonatally sensitized mice. J Virol. 2008;82(8):4115–24. Epub 2008/02/15. doi: JVI.02313-07 [pii] 10.1128/JVI.02313-07 18272579 PMC2293007

[9] Newell EW, Sigal N, Bendall SC, Nolan GP, Davis MM. Cytometry by time-of-flight shows combinatorial cytokine expression and virus-specific cell niches within a continuum of CD8+ T cell phenotypes. Immunity. 2012;36(1):142–52. 10.1016/j.immuni.2012.01.002 22265676 PMC3752833

[10] Billam P, Bonaparte KL, Liu J, Ruckwardt TJ, Chen M, Ryder AB, et al T Cell receptor clonotype influences epitope hierarchy in the CD8+ T cell response to respiratory syncytial virus infection. J Biol Chem. 2011;286(6):4829–41. 10.1074/jbc.M110.191437 21118816 PMC3039322

[11] Gallimore A, Dumrese T, Hengartner H, Zinkernagel RM, Rammensee HG. Protective immunity does not correlate with the hierarchy of virus-specific cytotoxic T cell responses to naturally processed peptides. J Exp Med. 1998;187(10):1647–57. Epub 1998/06/13. 9584143 10.1084/jem.187.10.1647 PMC2212291

[12] Frahm N, Kiepiela P, Adams S, Linde CH, Hewitt HS, Sango K, et al Control of human immunodeficiency virus replication by cytotoxic T lymphocytes targeting subdominant epitopes. Nat Immunol. 2006;7(2):173–8. Epub 2005/12/22. doi: ni1281 [pii] 10.1038/ni1281 . 16369537

[13] Hansen SG, Sacha JB, Hughes CM, Ford JC, Burwitz BJ, Scholz I, et al Cytomegalovirus vectors violate CD8+ T cell epitope recognition paradigms. Science. 2013;340(6135):1237874 10.1126/science.1237874 . 23704576 PMC3816976

[14] Rameix-Welti MA, Le Goffic R, Herve PL, Sourimant J, Remot A, Riffault S, et al Visualizing the replication of respiratory syncytial virus in cells and in living mice. Nat Commun. 2014;5:5104 10.1038/ncomms6104 . 25277263 PMC7091779

[15] Peebles RS Jr., Graham BS. Pathogenesis of respiratory syncytial virus infection in the murine model. Proc Am Thorac Soc. 2005;2(2):110–5. 10.1513/pats.200501-002AW 16113477 PMC2713314

[16] Graham BS, Perkins MD, Wright PF, Karzon DT. Primary respiratory syncytial virus infection in mice. J Med Virol. 1988;26(2):153–62. Epub 1988/10/01. . 3183639 10.1002/jmv.1890260207

[17] Rutigliano JA, Ruckwardt TJ, Martin JE, Graham BS. Relative dominance of epitope-specific CD8+ T cell responses in an F1 hybrid mouse model of respiratory syncytial virus infection. Virology. 2007;362(2):314–9. Epub 2007/02/06. doi: S0042-6822(06)00895-6 [pii] 10.1016/j.virol.2006.12.023 17275872 PMC1950131

[18] Simmons CP, Hussell T, Sparer T, Walzl G, Openshaw P, Dougan G. Mucosal delivery of a respiratory syncytial virus CTL peptide with enterotoxin-based adjuvants elicits protective, immunopathogenic, and immunoregulatory antiviral CD8+ T cell responses. J Immunol. 2001;166(2):1106–13. Epub 2001/01/06. . 11145691 10.4049/jimmunol.166.2.1106

[19] Ostler T, Davidson W, Ehl S. Virus clearance and immunopathology by CD8(+) T cells during infection with respiratory syncytial virus are mediated by IFN-gamma. Eur J Immunol. 2002;32(8):2117–23. Epub 2002/09/05. 10.1002/1521-4141(200208)32:8<2117::AID-IMMU2117>3.0.CO;2-C . 12209623

[20] Ruckwardt TJ, Luongo C, Malloy AM, Liu J, Chen M, Collins PL, et al Responses against a subdominant CD8+ T cell epitope protect against immunopathology caused by a dominant epitope. J Immunol. 2010;185(8):4673–80. 10.4049/jimmunol.1001606 20833834 PMC4144756

[21] Anderson KG, Sung H, Skon CN, Lefrancois L, Deisinger A, Vezys V, et al Cutting edge: intravascular staining redefines lung CD8 T cell responses. J Immunol. 2012;189(6):2702–6. 10.4049/jimmunol.1201682 22896631 PMC3436991

[22] Teijaro JR, Turner D, Pham Q, Wherry EJ, Lefrancois L, Farber DL. Cutting edge: Tissue-retentive lung memory CD4 T cells mediate optimal protection to respiratory virus infection. J Immunol. 2011;187(11):5510–4. 10.4049/jimmunol.1102243 22058417 PMC3221837

[23] Ruckwardt TJ, Malloy AM, Gostick E, Price DA, Dash P, McClaren JL, et al Neonatal CD8 T-cell hierarchy is distinct from adults and is influenced by intrinsic T cell properties in respiratory syncytial virus infected mice. PLoS Pathog. 7(12):e1002377 Epub 2011/12/07. 10.1371/journal.ppat.1002377 PPATHOGENS-D-11-01551 [pii]. 22144888 PMC3228797

[24] Coles RM, Mueller SN, Heath WR, Carbone FR, Brooks AG. Progression of armed CTL from draining lymph node to spleen shortly after localized infection with herpes simplex virus 1. J Immunol. 2002;168(2):834–8. Epub 2002/01/05. . 11777979 10.4049/jimmunol.168.2.834

[25] Hu G, Chen J. A genome-wide regulatory network identifies key transcription factors for memory CD8(+) T-cell development. Nat Commun. 2013;4:2830 10.1038/ncomms3830 24335726 PMC3999894

[26] Farber DL, Yudanin NA, Restifo NP. Human memory T cells: generation, compartmentalization and homeostasis. Nat Rev Immunol. 2014;14(1):24–35. 10.1038/nri3567 24336101 PMC4032067

[27] Best JA, Blair DA, Knell J, Yang E, Mayya V, Doedens A, et al Transcriptional insights into the CD8(+) T cell response to infection and memory T cell formation. Nat Immunol. 2013;14(4):404–12. 10.1038/ni.2536 23396170 PMC3689652

[28] Paley MA, Kroy DC, Odorizzi PM, Johnnidis JB, Dolfi DV, Barnett BE, et al Progenitor and terminal subsets of CD8+ T cells cooperate to contain chronic viral infection. Science. 2012;338(6111):1220–5. 10.1126/science.1229620 23197535 PMC3653769

[29] Wherry EJ. T cell exhaustion. Nat Immunol. 2011;12(6):492–9. . 21739672 10.1038/ni.2035

[30] Pagan JK, Arnold J, Hanchard KJ, Kumar R, Bruno T, Jones MJ, et al A novel corepressor, BCoR-L1, represses transcription through an interaction with CtBP. J Biol Chem. 2007;282(20):15248–57. Epub 2007/03/24. doi: M700246200 [pii] 10.1074/jbc.M700246200 . 17379597

[31] Huynh KD, Fischle W, Verdin E, Bardwell VJ. BCoR, a novel corepressor involved in BCL-6 repression. Genes Dev. 2000;14(14):1810–23. Epub 2000/07/18. 10898795 PMC316791

[32] Krona C, Ejeskar K, Caren H, Abel F, Sjoberg RM, Martinsson T. A novel 1p36.2 located gene, APITD1, with tumour-suppressive properties and a putative p53-binding domain, shows low expression in neuroblastoma tumours. Br J Cancer. 2004;91(6):1119–30. Epub 2004/08/26. 10.1038/sj.bjc.6602083 6602083 [pii]. 15328517 PMC2747717

[33] Boyd JM, Gallo GJ, Elangovan B, Houghton AB, Malstrom S, Avery BJ, et al Bik, a novel death-inducing protein shares a distinct sequence motif with Bcl-2 family proteins and interacts with viral and cellular survival-promoting proteins. Oncogene. 1995;11(9):1921–8. Epub 1995/11/02. . 7478623

[34] Ghavami S, Hashemi M, Ande SR, Yeganeh B, Xiao W, Eshraghi M, et al Apoptosis and cancer: mutations within caspase genes. J Med Genet. 2009;46(8):497–510. Epub 2009/06/10. doi: jmg.2009.066944 [pii] 10.1136/jmg.2009.066944 . 19505876

[35] Prasad KV, Ao Z, Yoon Y, Wu MX, Rizk M, Jacquot S, et al CD27, a member of the tumor necrosis factor receptor family, induces apoptosis and binds to Siva, a proapoptotic protein. Proc Natl Acad Sci U S A. 1997;94(12):6346–51. Epub 1997/06/10. 9177220 10.1073/pnas.94.12.6346 PMC21052

[36] Wu M, Xu LG, Li X, Zhai Z, Shu HB. AMID, an apoptosis-inducing factor-homologous mitochondrion-associated protein, induces caspase-independent apoptosis. J Biol Chem. 2002;277(28):25617–23. Epub 2002/05/01. 10.1074/jbc.M202285200 M202285200 [pii]. . 11980907

[37] Shibahara K, Asano M, Ishida Y, Aoki T, Koike T, Honjo T. Isolation of a novel mouse gene MA-3 that is induced upon programmed cell death. Gene. 1995;166(2):297–301. Epub 1995/12/12. doi: 0378111995006079 [pii]. . 8543179 10.1016/0378-1119(95)00607-9

[38] Bergametti F, Denier C, Labauge P, Arnoult M, Boetto S, Clanet M, et al Mutations within the programmed cell death 10 gene cause cerebral cavernous malformations. Am J Hum Genet. 2005;76(1):42–51. Epub 2004/11/16. doi: S0002-9297(07)62542-7 [pii] 10.1086/426952 15543491 PMC1196432

[39] Satterwhite E, Sonoki T, Willis TG, Harder L, Nowak R, Arriola EL, et al The BCL11 gene family: involvement of BCL11A in lymphoid malignancies. Blood. 2001;98(12):3413–20. Epub 2001/11/24. . 11719382 10.1182/blood.v98.12.3413

[40] Baron BW, Anastasi J, Thirman MJ, Furukawa Y, Fears S, Kim DC, et al The human programmed cell death-2 (PDCD2) gene is a target of BCL6 repression: implications for a role of BCL6 in the down-regulation of apoptosis. Proc Natl Acad Sci U S A. 2002;99(5):2860–5. Epub 2002/02/21. 10.1073/pnas.042702599 042702599 [pii]. 11854457 PMC122438

[41] Hitoshi Y, Lorens J, Kitada SI, Fisher J, LaBarge M, Ring HZ, et al Toso, a cell surface, specific regulator of Fas-induced apoptosis in T cells. Immunity. 1998;8(4):461–71. Epub 1998/05/20. doi: S1074-7613(00)80551-8 [pii]. . 9586636 10.1016/s1074-7613(00)80551-8

[42] Hansen SG, Piatak M Jr., Ventura AB, Hughes CM, Gilbride RM, Ford JC, et al Immune clearance of highly pathogenic SIV infection. Nature. 2013;502(7469):100–4. 10.1038/nature12519 24025770 PMC3849456

[43] Aung S, Rutigliano JA, Graham BS. Alternative mechanisms of respiratory syncytial virus clearance in perforin knockout mice lead to enhanced disease. J Virol. 2001;75(20):9918–24. Epub 2001/09/18. 10.1128/JVI.75.20.9918–9924.2001 11559824 PMC114563

[44] Chang J, Braciale TJ. Respiratory syncytial virus infection suppresses lung CD8+ T-cell effector activity and peripheral CD8+ T-cell memory in the respiratory tract. Nat Med. 2002;8(1):54–60. Epub 2002/01/12. 10.1038/nm0102-54 nm0102-54 [pii]. . 11786907

[45] Schenkel JM, Fraser KA, Vezys V, Masopust D. Sensing and alarm function of resident memory CD8(+) T cells. Nat Immunol. 2013;14(5):509–13. 10.1038/ni.2568 23542740 PMC3631432

[46] Knudson CJ, Weiss KA, Hartwig SM, Varga SM. The pulmonary localization of virus-specific T lymphocytes is governed by the tissue tropism of infection. J Virol. 2014;88(16):9010–6. 10.1128/JVI.00329-14 24899187 PMC4136240

[47] Alexander-Miller MA, Leggatt GR, Berzofsky JA. Selective expansion of high- or low-avidity cytotoxic T lymphocytes and efficacy for adoptive immunotherapy. Proc Natl Acad Sci U S A. 1996;93(9):4102–7. Epub 1996/04/30. 8633023 10.1073/pnas.93.9.4102 PMC39494

[48] Messaoudi I, Guevara Patino JA, Dyall R, LeMaoult J, Nikolich-Zugich J. Direct link between mhc polymorphism, T cell avidity, and diversity in immune defense. Science. 2002;298(5599):1797–800. Epub 2002/12/03. 10.1126/science.1076064 298/5599/1797 [pii]. . 12459592

[49] Billam P, Bonaparte KL, Liu J, Ruckwardt TJ, Chen M, Ryder AB, et al T Cell receptor clonotype influences epitope hierarchy in the CD8+ T cell response to respiratory syncytial virus infection. J Biol Chem. 286(6):4829–41. Epub 2010/12/02. doi: M110.191437 [pii] 10.1074/jbc.M110.191437 21118816 PMC3039322

[50] Turner SJ, La Gruta NL, Kedzierska K, Thomas PG, Doherty PC. Functional implications of T cell receptor diversity. Curr Opin Immunol. 2009;21(3):286–90. 10.1016/j.coi.2009.05.004 19524428 PMC2706259

[51] Hansen SG, Vieville C, Whizin N, Coyne-Johnson L, Siess DC, Drummond DD, et al Effector memory T cell responses are associated with protection of rhesus monkeys from mucosal simian immunodeficiency virus challenge. Nat Med. 2009;15(3):293–9. Epub 2009/02/17. doi: nm.1935 [pii] 10.1038/nm.1935 19219024 PMC2720091

[52] Buchholz VR, Flossdorf M, Hensel I, Kretschmer L, Weissbrich B, Graf P, et al Disparate individual fates compose robust CD8+ T cell immunity. Science. 2013;340(6132):630–5. 10.1126/science.1235454 . 23493420

[53] Obar JJ, Khanna KM, Lefrancois L. Endogenous naive CD8+ T cell precursor frequency regulates primary and memory responses to infection. Immunity. 2008;28(6):859–69. Epub 2008/05/24. doi: S1074-7613(08)00200-8 [pii] 10.1016/j.immuni.2008.04.010 18499487 PMC2836785

[54] Kotturi MF, Scott I, Wolfe T, Peters B, Sidney J, Cheroutre H, et al Naive precursor frequencies and MHC binding rather than the degree of epitope diversity shape CD8+ T cell immunodominance. J Immunol. 2008;181(3):2124–33. Epub 2008/07/22. doi: 181/3/2124 [pii]. 18641351 10.4049/jimmunol.181.3.2124 PMC3319690

[55] Labrecque N, Whitfield LS, Obst R, Waltzinger C, Benoist C, Mathis D. How much TCR does a T cell need? Immunity. 2001;15(1):71–82. . 11485739 10.1016/s1074-7613(01)00170-4

[56] Leignadier J, Rooney J, Daudelin JF, Labrecque N. Lowering TCR expression on naive CD8+ T cells does not affect memory T-cell differentiation. Immunol Cell Biol. 2011;89(2):322–5. 10.1038/icb.2010.80 . 20585337

[57] de Bree GJ, van Leeuwen EM, Out TA, Jansen HM, Jonkers RE, van Lier RA. Selective accumulation of differentiated CD8+ T cells specific for respiratory viruses in the human lung. J Exp Med. 2005;202(10):1433–42. Epub 2005/11/23. doi: jem.20051365 [pii] 10.1084/jem.20051365 16301748 PMC2212987

[58] Liu J, Ruckwardt TJ, Chen M, Nicewonger JD, Johnson TR, Graham BS. Epitope-specific regulatory CD4 T cells reduce virus-induced illness while preserving CD8 T-cell effector function at the site of infection. J Virol. 84(20):10501–9. Epub 2010/08/06. doi: JVI.00963-10 [pii] 10.1128/JVI.00963-10 20686045 PMC2950556

[59] Erickson JJ, Gilchuk P, Hastings AK, Tollefson SJ, Johnson M, Downing MB, et al Viral acute lower respiratory infections impair CD8+ T cells through PD-1. J Clin Invest. 2012;122(8):2967–82. 10.1172/JCI62860 22797302 PMC3408742

[60] Telcian AG, Laza-Stanca V, Edwards MR, Harker JA, Wang H, Bartlett NW, et al RSV-induced bronchial epithelial cell PD-L1 expression inhibits CD8+ T cell nonspecific antiviral activity. J Infect Dis. 203(1):85–94. Epub 2010/12/15. doi: jiq020 [pii] 10.1093/infdis/jiq020 21148500 PMC3086441

[61] Hogan RJ, Usherwood EJ, Zhong W, Roberts AA, Dutton RW, Harmsen AG, et al Activated antigen-specific CD8+ T cells persist in the lungs following recovery from respiratory virus infections. J Immunol. 2001;166(3):1813–22. Epub 2001/02/13. . 11160228 10.4049/jimmunol.166.3.1813

[62] De Jong R, Brouwer M, Hooibrink B, Van der Pouw-Kraan T, Miedema F, Van Lier RA. The CD27- subset of peripheral blood memory CD4+ lymphocytes contains functionally differentiated T lymphocytes that develop by persistent antigenic stimulation in vivo. Eur J Immunol. 1992;22(4):993–9. Epub 1992/04/01. 10.1002/eji.1830220418 . 1348033

[63] Linsley PS, Bradshaw J, Urnes M, Grosmaire L, Ledbetter JA. CD28 engagement by B7/BB-1 induces transient down-regulation of CD28 synthesis and prolonged unresponsiveness to CD28 signaling. J Immunol. 1993;150(8 Pt 1):3161–9. Epub 1993/04/15. . 7682233

[64] Gamadia LE, van Leeuwen EM, Remmerswaal EB, Yong SL, Surachno S, Wertheim-van Dillen PM, et al The size and phenotype of virus-specific T cell populations is determined by repetitive antigenic stimulation and environmental cytokines. J Immunol. 2004;172(10):6107–14. Epub 2004/05/07. . 15128796 10.4049/jimmunol.172.10.6107

[65] Kaech SM, Tan JT, Wherry EJ, Konieczny BT, Surh CD, Ahmed R. Selective expression of the interleukin 7 receptor identifies effector CD8 T cells that give rise to long-lived memory cells. Nat Immunol. 2003;4(12):1191–8. Epub 2003/11/20. 10.1038/ni1009 ni1009 [pii]. . 14625547

[66] van Leeuwen EM, de Bree GJ, Remmerswaal EB, Yong SL, Tesselaar K, ten Berge IJ, et al IL-7 receptor alpha chain expression distinguishes functional subsets of virus-specific human CD8+ T cells. Blood. 2005;106(6):2091–8. Epub 2005/06/11. doi: 2005-02-0449 [pii] 10.1182/blood-2005-02-0449 . 15947093

[67] Melenhorst JJ, Scheinberg P, Chattopadhyay PK, Lissina A, Gostick E, Cole DK, et al Detection of low avidity CD8(+) T cell populations with coreceptor-enhanced peptide-major histocompatibility complex class I tetramers. Journal of immunological methods. 2008;338(1–2):31–9. 10.1016/j.jim.2008.07.008 18675271 PMC2714739

