# Supplementary figures and images for: A Numerically Subdominant CD8 T Cell Response to Matrix Protein of Respiratory Syncytial Virus Controls Infection with Limited Immunopathology
Source: PLoS Pathog. 2016 Mar 4;12(3):e1005486. doi: 10.1371/journal.ppat.1005486 (PMC4778879; doi:10.1371/journal.ppat.1005486)

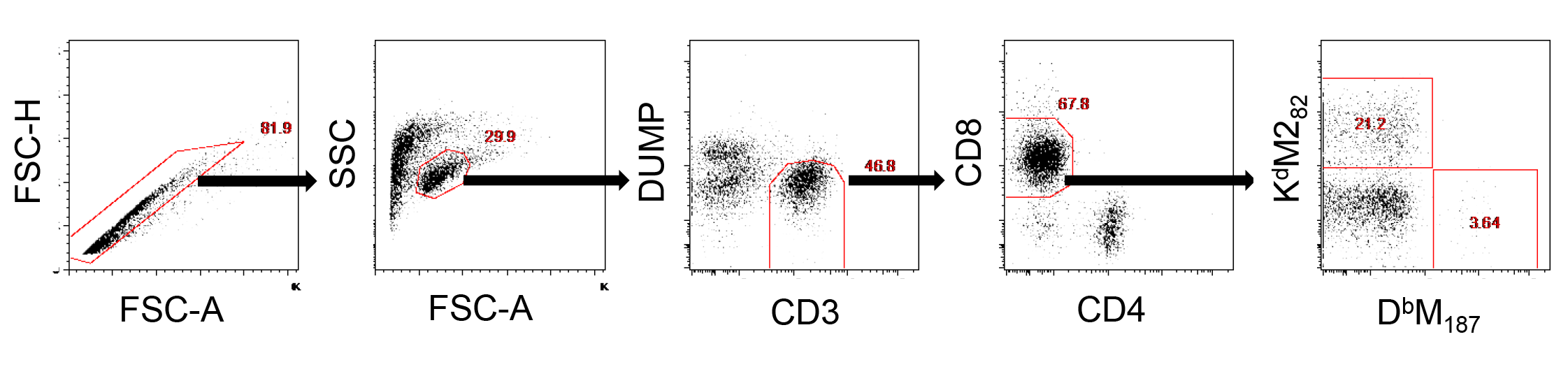

Supplement: S1 Fig — Lymphocytes isolated from the lung and spleen of RSV-infected mice at 7 dpi were stained for CD3, CD4 and CD8 lineage differentiation and pMHC-specificities. Dot plots represent strategies to identify DbM187 and KdM282 T cells, by sequentially gating on singlets, lymphocytes, CD3(+) Dump(-), CD8(+) CD4(-), and pMHC(+) populations. The Dump channel included Violet fluorescent reactive dye, fluorochrom-conjugated anti-CD16/32 and anti-CD19 to exclude dead cells, monocytes, B cells, and other non-T cells. Dot plots represent 5 independent experiments (n = 5/group/experiment). (TIF) [file ppat.1005486.s001.tif]

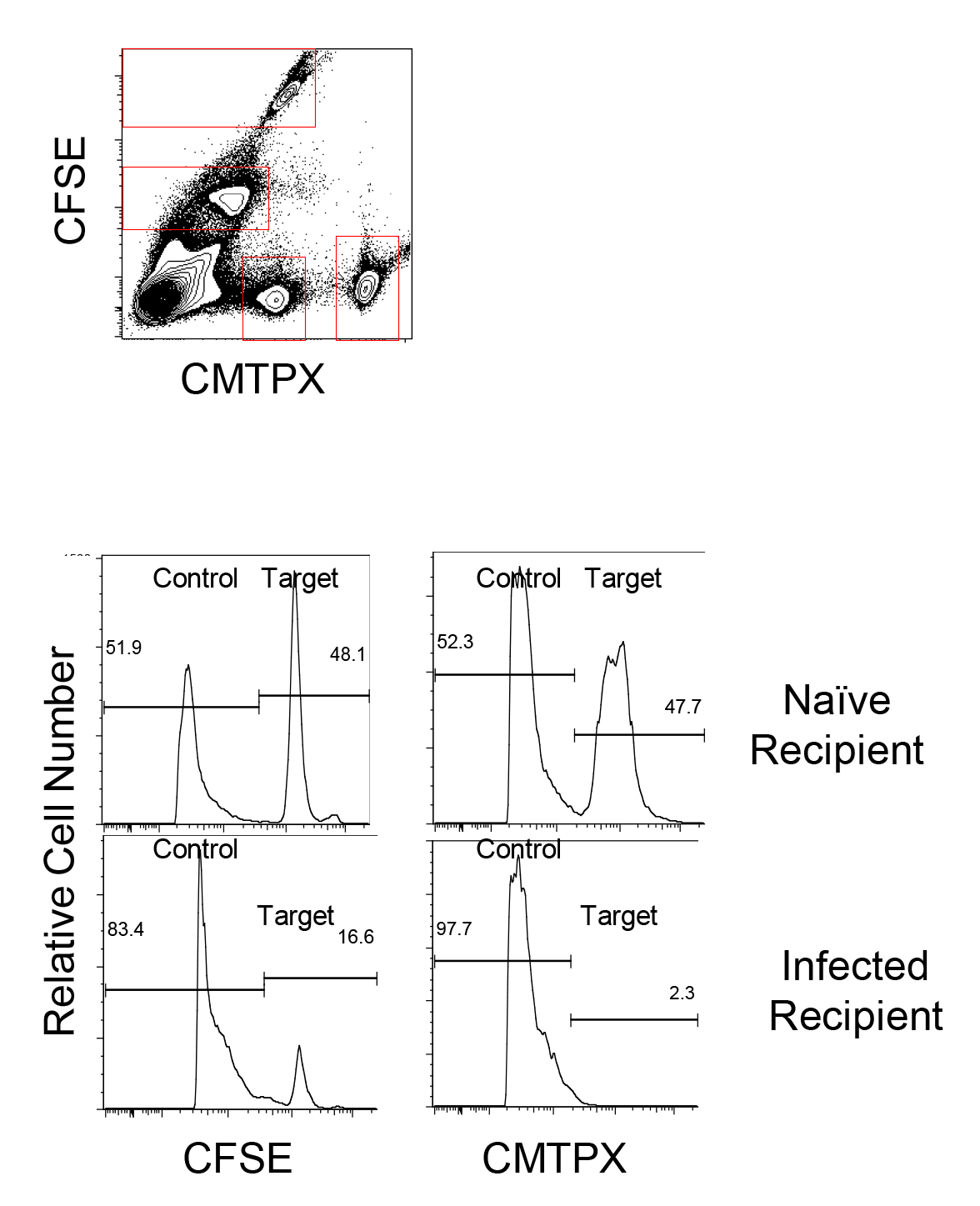

Supplement: S2 Fig — After adopting peptide-loaded and fluorochrome-labeled targets and controls, the recipient’s lung and spleen cells were isolated 3 hours later and analyzed by flow cytometry. The M187 and M282 peptide-loaded targets were identified by high intensity of CFSE and CMTPX respectively; the OVA257 peptide-loaded controls were identified by lower intensity of CFSE and CMTPX and used as controls for the same fluorochrome-labeled targets respectively. Histograms show the proportion of cells with distinct fluorochrome labeling and intensity recovered from infected or naïve mice. Dot plots and histograms represent 5 independent experiments (n = 5/group/experiment). (TIF) [file ppat.1005486.s002.tif]

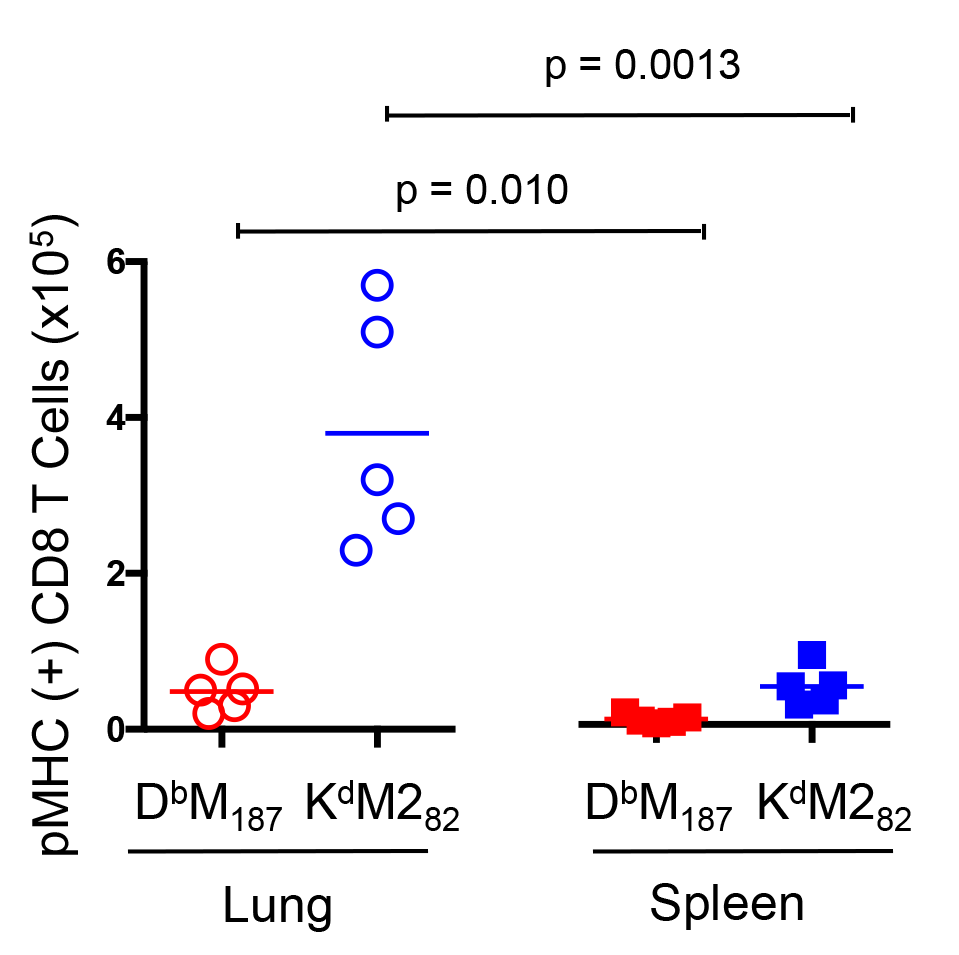

Supplement: S3 Fig — The DbM187 and KdM282 T cells from lung and spleen of infected mice at 7 dpi were quantitatively assessed by flow cytometry. Cell counts were calculated based on total cell counts in the lung and spleen cell preparations and the frequency of individual subsets. Data are shown as mean with independent data point and are compared by Student’s t-test. Data represent 5 independent experiments (n = 5/group/experiment). Each symbol represents one mouse. (TIF) [file ppat.1005486.s003.tif]

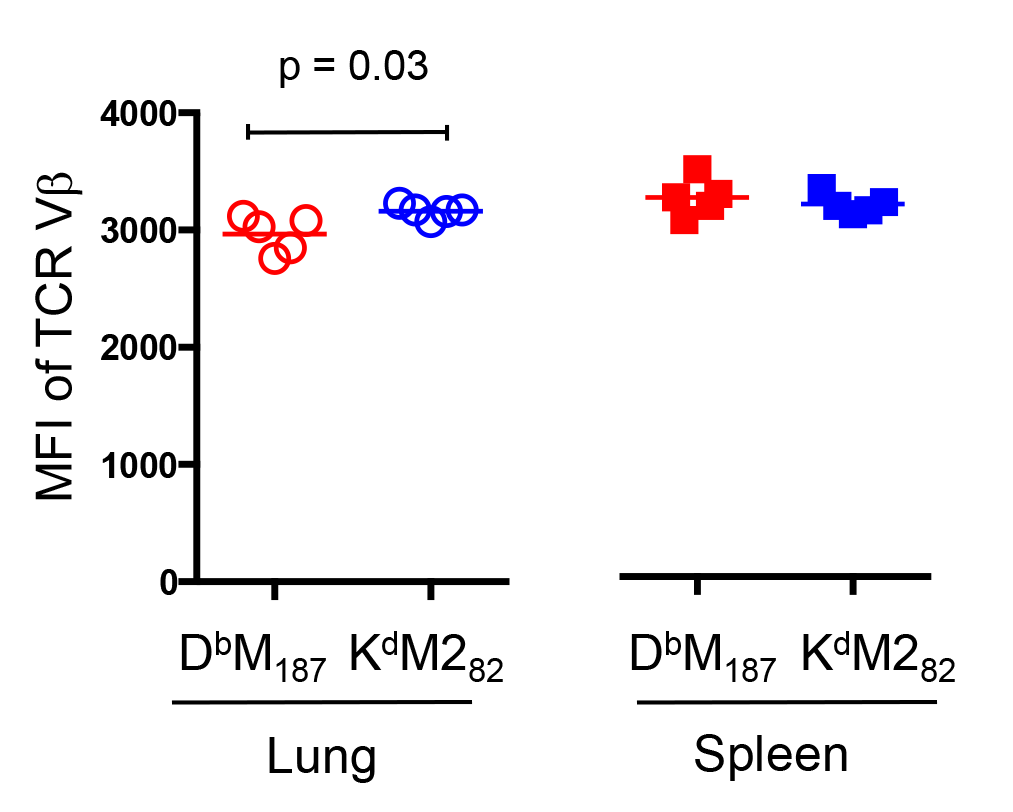

Supplement: S4 Fig — Lymphocytes isolated from the lung and spleen of RSV-infected mice at 7 dpi were stained for CD3, CD4 and CD8 lineage differentiation and pMHC-specificities. The expression of TCR on epitope-specific CD8 T cells was studied using fluorescence-conjugated anti-TCR Vβ (pan Vβ) monoclonal antibody, and the relative level of expression was measured by the medium fluorescence intensity (MFI). Data are shown as mean with independent data point and are compared by Student’s t-test. Data represent 2 independent experiments (n = 5/group/experiment). Each symbol represents one mouse. (TIF) [file ppat.1005486.s004.tif]

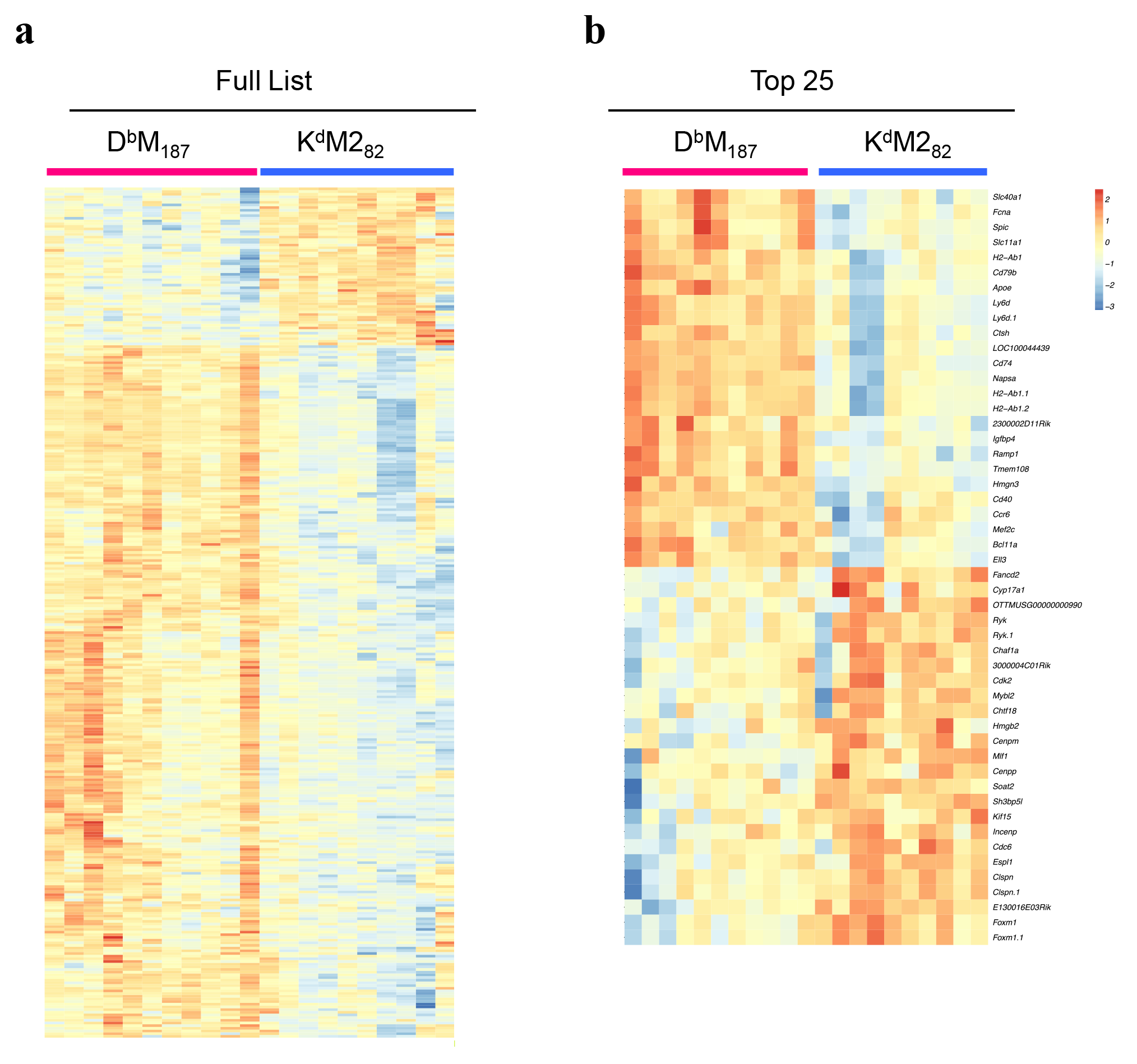

Supplement: S5 Fig — The DbM187, KdM282 and bulk CD8 T cells were sorted from spleen by FACS at 7 dpi and studied for transcriptional expression of genes associated to immune responses. The quantitative gene expression were analyzed and normalized. Relative expression was calculated and presented as Log2 Fold Change (Log2FC). The Log2FC = Log2 KdM282 –Log2 DbM187. Positive values indicate gene expression was up-regulated in the KdM282 subset, while negative values indicate gene expression was up-regulated in the DbM187 subset. (a) All gene expressions with Log2 FC > ±1.3. (b) Top 25 up-regulated expressions among (a) in each subset. (c) Up-regulated expressions of genes in (a) related to MHC class I molecule and clone expansion. Each column represents data from one mouse. (TIF) [file ppat.1005486.s005.tif]

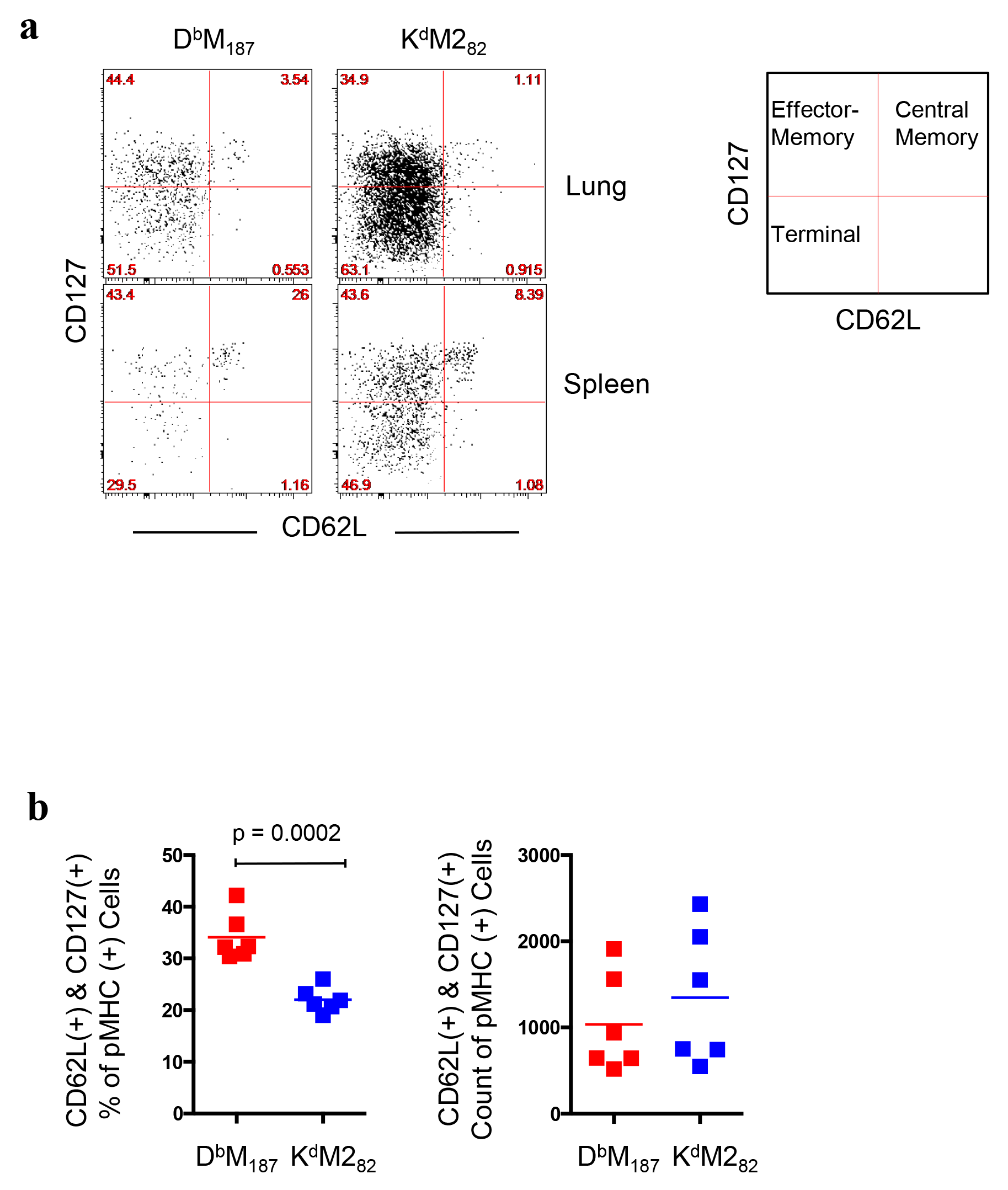

Supplement: S6 Fig — Lymphocytes were isolated from the lung and spleen of infected mice at 7dpi and studied for expression of CD62L and CD127 with flow cytometry. Dot plots show the gating strategy and represent 5 independent experiments (n = 5/group/experiment). (b) Lymphocytes were isolated from the lung and spleen of mice at 8 weeks post infection. The DbM187 and KdM282 T cells were studied for their frequency, count, and expression of CD62L and CD127 with flow cytometry. Data are shown as individual data points with horizontal bar representing the mean, and are compared by Student’s t-test. Data represent 2 independent experiments (n = 5/group/experiment). Each symbol represents one mouse. (TIF) [file ppat.1005486.s006.tif]

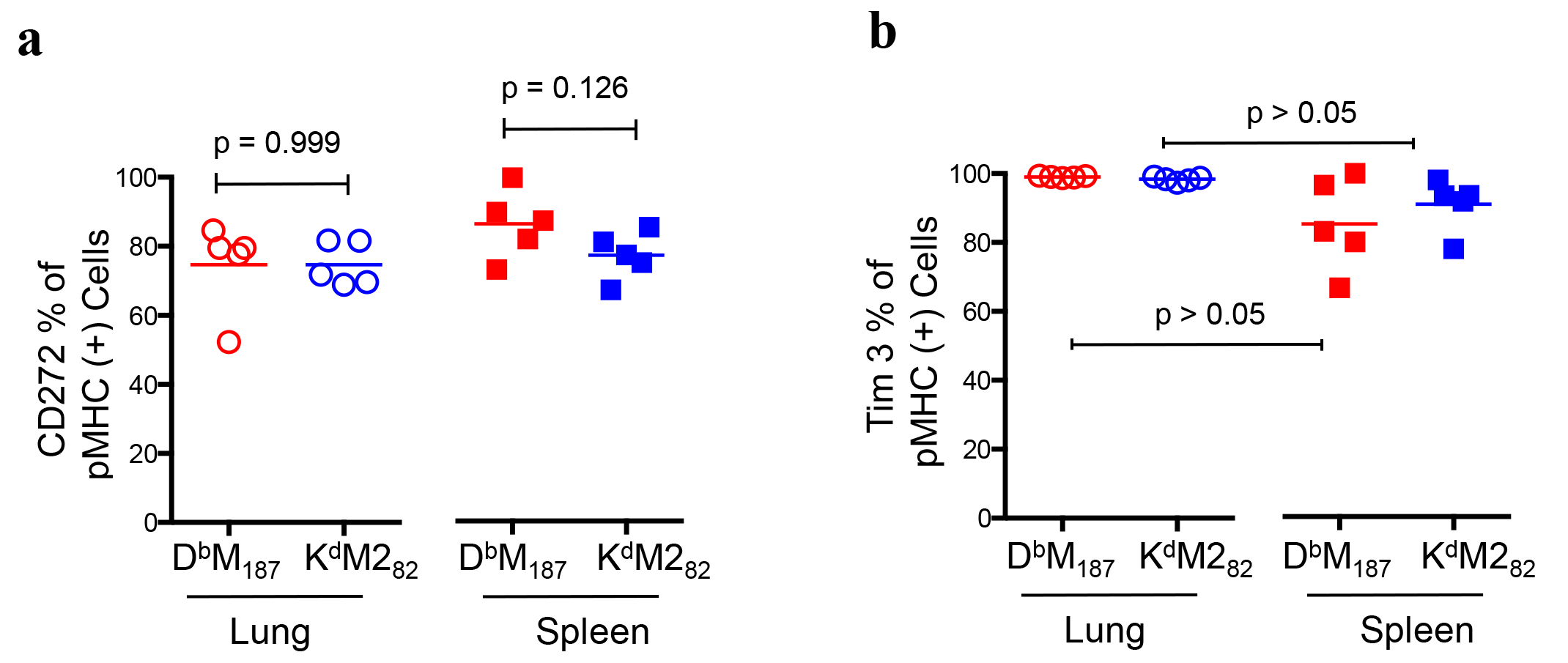

Supplement: S7 Fig — Lymphocytes were isolated from the lung and spleen of infected mice at 7dpi and studied for expression of (a) CD272 and (b) Tim 3 with flow cytometry. The frequencies are shown as mean with independent data point and compared by Student’s t-test. Data represent 5 independent experiments (n = 5/group/experiment). Each symbol represents one mouse. (TIF) [file ppat.1005486.s007.tif]

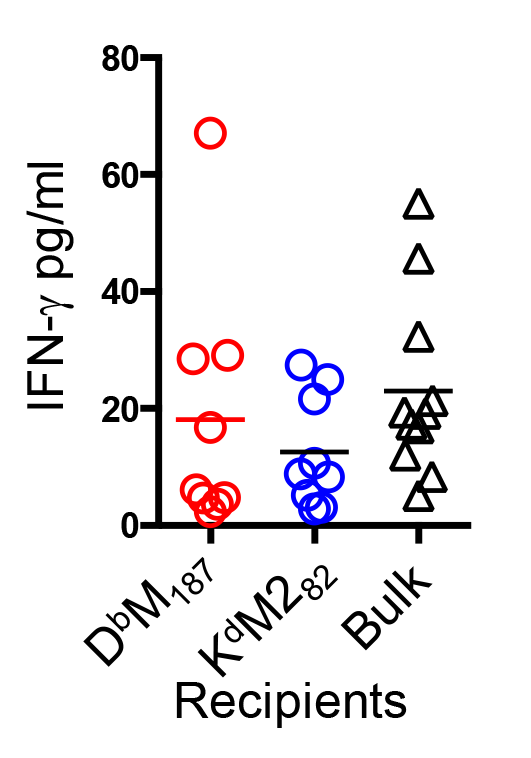

Supplement: S8 Fig — Lung supernatant described in Fig 7B were collected and frozen saved. INF-γ level was assessed using LEGENDplex (BioLegend), a bead-based commercially available immunoassay service. The concentration was calculated referring to standard series. Each symbol represents one mouse (n = 11). (TIF) [file ppat.1005486.s008.tif]

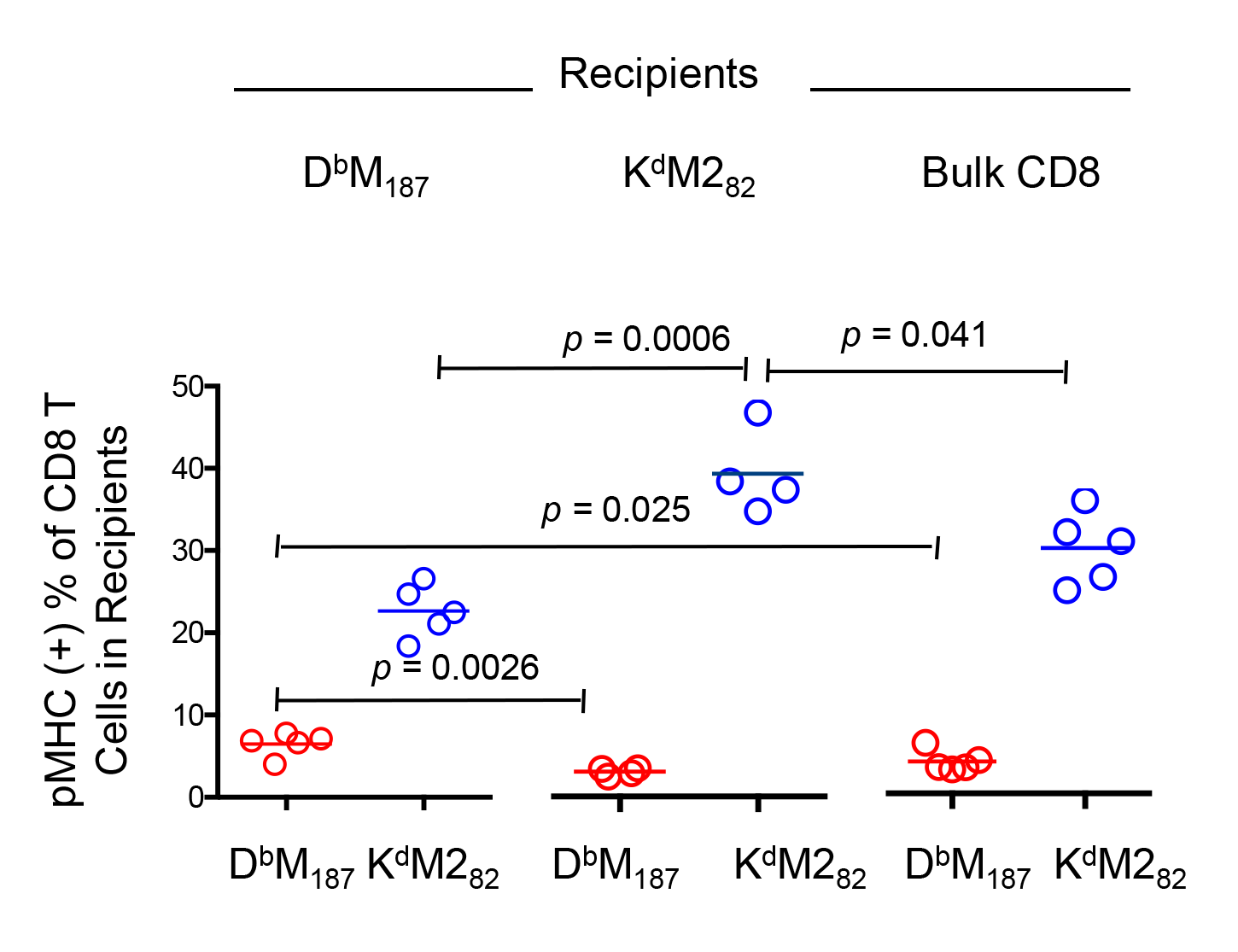

Supplement: S9 Fig — After virus challenge, the recipients that adopted DbM187, KdM282 and bulk CD8 T cells were evaluated for DbM187 and KdM282 T cell frequencies in the right lung at 7 dpi with flow cytometry. The frequencies are shown as mean with independent data point and compared by Student’s t-test. Data represent 3 independent experiments (n = 4 or 5/group/experiment). Each symbol represents one mouse. (TIF) [file ppat.1005486.s009.tif]

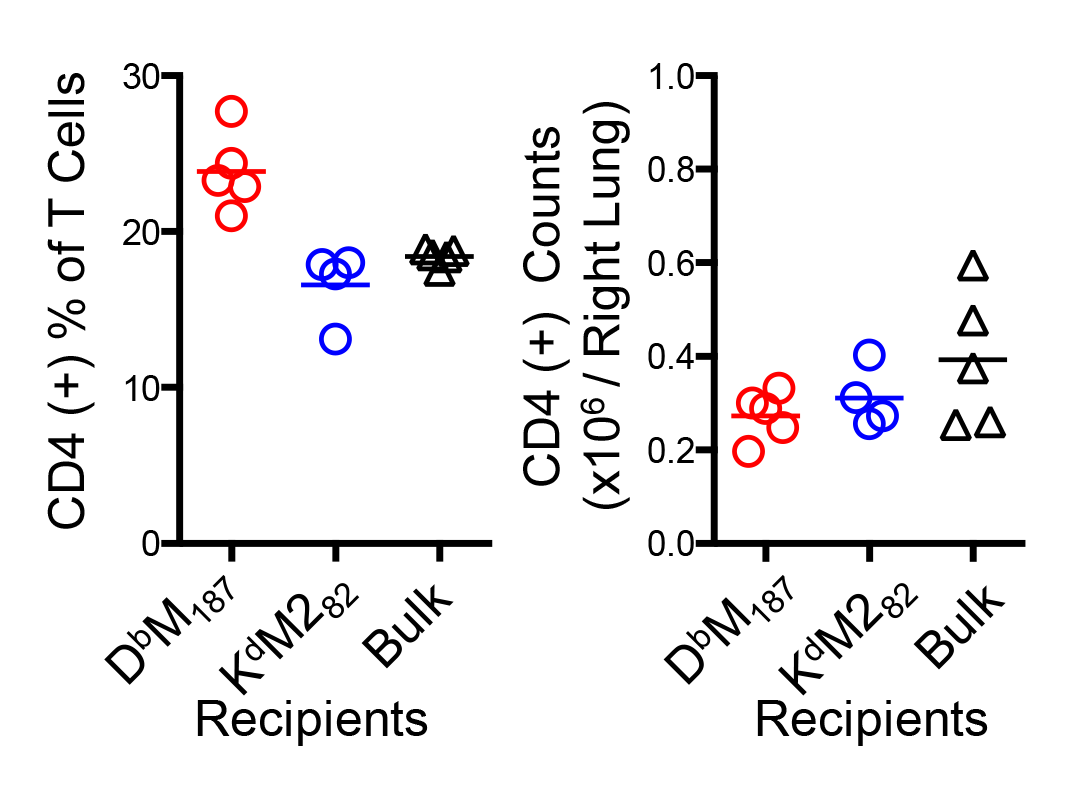

Supplement: S10 Fig — The DbM187, KdM282 and bulk CD8 T cell recipients were challenged with RSV. Inflammatory cells were assessed at 7 dpi with flow cytometry. The absolute number and frequency of CD4 (+) cells are expressed as mean with independent data point and are compared by Student’s t-test. Data represent 3 independent experiments (n = 5/group/experiment). Each symbol represents one mouse. (TIF) [file ppat.1005486.s010.tif]

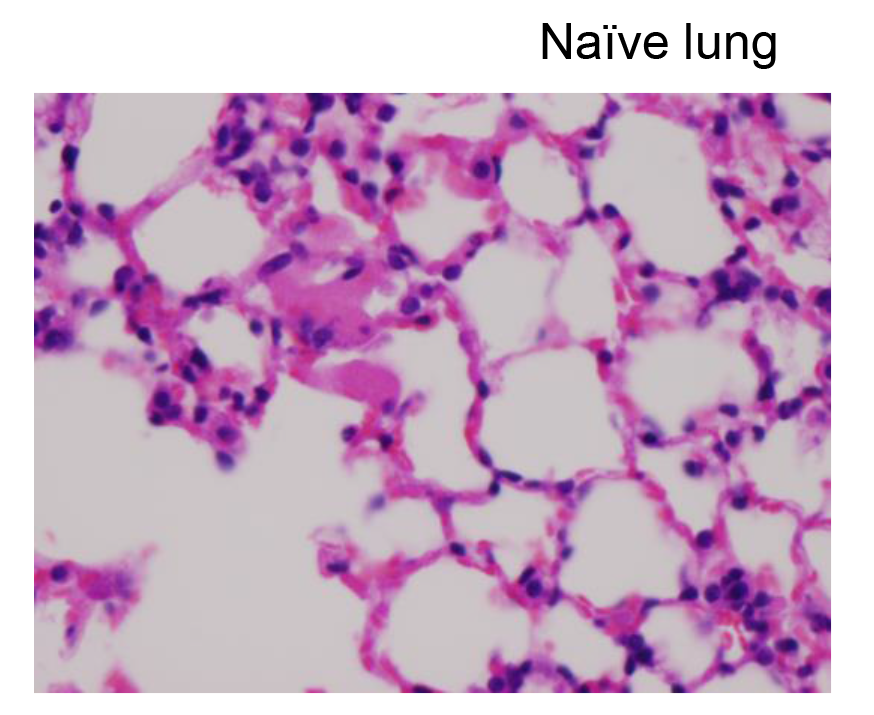

Supplement: S11 Fig — Left lung were isolated from naïve mice and studied for the histology of lung structure. Sections were examined under light microscope by independent blinded investigators. Histology study represents 3 independent experiments (n = 5). (TIF) [file ppat.1005486.s011.tif]
